# Supplementary material for: Transcriptomic analysis to identify genes associated with selective hippocampal vulnerability in Alzheimer’s disease
Source: Nat Commun. 2021 Apr 19;12:2311. doi: 10.1038/s41467-021-22399-3 (PMC8055900; doi:10.1038/s41467-021-22399-3)
Supplement: Supplementary file 1 — Supplementary Information [file 41467_2021_22399_MOESM1_ESM.pdf]

Supplementary Information for Transcriptomic analysis to identify genes associated with selective hippocampal vulnerability in Alzheimer's disease

Angela M. Crist[1], Kelly M. Hinkle[1], Xue Wang[2], Christina M. Moloney[1], Billie J. Matchett[1], Sydney A. Labuzan[1], Isabelle Frankenhauser[1,3], Nkem O. Azu[1], Amanda M. Liesinger[1], Elizabeth R. Lesser[2], Daniel J. Serie[2], Zachary S. Quicksall[2], Tulsi A. Patel Ph.D[1], Troy P. Carnwath[1], Michael DeTure[1], Xiaojia Tang[4], Ronald C. Petersen[5], Ranjan Duara[6], Neill R. Graff-Radford[7], Mariet Allen[1], Minerva M. Carrasquillo[1], Hu Li[8], Owen A. Ross[1], Nilufer Ertekin-Taner[1,7], Dennis W. Dickson[1], Yan W. Asmann[2], Rickey E. Carter[2], and Melissa E. Murray[1]*

[1] Department of Neuroscience, Mayo Clinic, Jacksonville, FL, USA
[2] Department of Health Sciences Research, Mayo Clinic, Jacksonville, FL, USA
[3] Paracelsus Medical Private University, Salzburg, Austria
[4] Department of Health Sciences Research, Mayo Clinic, Rochester, MN, USA
[5] Department of Neurology, Mayo Clinic, Rochester, MN, USA
[6] Wien Center for Alzheimer's Disease and Memory Disorders, Mount Sinai Medical Center, Miami Beach, FL, USA
[7] Department of Neurology, Mayo Clinic, Jacksonville, FL, USA
[8] Department of Molecular Pharmacology and Experimental Therapeutics, Mayo Clinic, Rochester, MN, USA

*Corresponding author: murray.melissa@mayo.edu

Characteristic	Controls (n=15)	AD neuropathologic subtypes (n=40)			AD specific p-value
		HpSp AD (n=10)	Typical AD (n=20)	Limbic predominant AD (n=10)	
Males (% total of AD type)	7/15 (47%)	4/10 (40%)	7/20 (35%)	4/10 (40%)	0.95
APOE ε4, %	1/15 (6.7%)	3/10 (30%)	8/20 (40%)	4/10 (40%)	0.85
**Clinical findings**					
Age at onset, yr.	NA (NA, NA)	66 (59, 69)	74 (70, 80)	79 (73, 80)	0.006
Disease duration, yr.	NA (NA, NA)	8.7 (7.3, 8.9)	7.7 (6.2, 10)	9.0 (6.9, 10)	0.76
Final MMSE, points/yr.	29 (28, 30)	13 (6.5, 16)	14 (12, 19)	14 (7.0, 21)	0.68
**Postmortem findings**					
Age at death, yr.	84 (80, 90)	73 (68, 76)	81 (78, 88)	87 (82, 89)	0.003
Braak tangle stage	II (I, III)	VI (V, VI)	VI (V, VI)	VI (V, VI)	0.95
Thal amyloid phase	0 (0, 1)	5 (5, 5)	5 (4, 5)	5 (5, 5)	0.38
Early tangle marker (CP13), %	0.22 (0.10, 1.5)	12 (8.2, 14)	12 (9.9, 17)	16 (13, 26)	0.11
Mature tangle marker (Ab39), %	0.040 (0.030, 0.080)	0.22 (0.19, 0.43)	0.27 (0.20, 0.37)	0.53 (0.44, 0.76)	0.018
Pan-Aβ marker (33.1.1), %	0.20 (0.080, 0.33)	0.56 (0.38, 0.81)	0.56 (0.43, 1.1)	0.50 (0.47, 0.60)	0.94
Astroglial marker (GFAP), %	29 (20, 36)	34 (22, 39)	36 (31, 47)	36 (32, 50)	0.34
Activated microglial marker (CD68), %	0.10 (0.060, 0.20)	0.24 (0.17, 0.37)	0.32 (0.22, 0.38)	0.51 (0.37, 0.63)	0.095
Endothelial cell marker (CD34), %	0.72 (0.50, 0.81)	0.52 (0.34, 0.64)	0.65 (0.37, 0.87)	0.87 (0.72, 0.93)	0.041

Supplementary Fig. 1 | RNA-Seq cohort characteristics. Table contains demographic, clinical, and neuropathologic characteristics of the RNA-Seq cohort. Data are presented as: sample size (percentage) or median (25th percentile, 75th percentile). Digital pathology measures are presented as percentage burden of immunopositive staining. Acronyms: AD=Alzheimer's disease, HpSp=hippocampal sparing AD, MMSE=Mini Mental State Examination, n=sample size, NFT=neurofibrillary tangle, yr=years. Note: Normal controls were not included in two-sided Kruskal Wallis Rank Sum Test to enable interpretation of p-values specific to group-wise comparisons among AD subtypes.

Corresponding figure	Description	Control vs HpSp AD	Control vs Typical AD	Control vs Limbic AD	HpSp AD vs Typical AD	HpSp AD vs Limbic AD	Typical AD vs Limbic AD
Fig. 3f	*SIRT1* levels from RNA-Seq	0.31	**0.034**	**0.0029**	0.38	**0.015**	0.12
Fig. 3g	*PSEN2* levels from RNA-Seq	0.37	**0.011**	**0.004**	0.19	0.11	0.68
Fig. 5a	*SERPINA5* levels from RNA-Seq	**0.001**	**<0.001**	**0.004**	0.56	0.58	0.81
Fig. 5b	*SERPINA5* levels from NanoString	**<0.001**	**<0.001**	**<0.001**	0.9	**0.023**	**0.011**
Fig. 5d	CA1 *SERPINA5* burden	**<0.001**	**<0.001**	**<0.001**	**0.026**	0.076	0.99
Fig. 5d	Subiculum *SERPINA5* burden	**<0.001**	**<0.001**	**<0.001**	0.096	**0.023**	0.64
Fig. 5d	Superior temporal *SERPINA5* burden	**<0.001**	**<0.001**	**<0.001**	0.9	**0.006**	0.14
Fig. 5d	Inferior temporal *SERPINA5* burden	**<0.001**	**<0.001**	**<0.001**	0.24	**<0.001**	**0.002**
Fig. 5d	Mid-frontal *SERPINA5* burden	**<0.001**	**<0.001**	**<0.001**	0.056	**<0.001**	**<0.001**

Supplementary Fig. 2 | Pair-wise comparisons of all box plots and violin plots. Statistical analyses from two-sided Wilcoxon Rank Sum tests showing pair-wise comparisons of all six groups from graphs throughout the manuscript. All statistically significant values (i.e. $p<0.05$) are bolded. Acronyms: AD=Alzheimer's disease, HpSp=hippocampal sparing, Limbic=Limbic predominant AD, RNA-Seq=RNA sequencing.

Gene selection	Phenotype derived	Genes	Transcript level associations with neuropathologic measures (false discovery rate<0.25, p-value<0.05)										Genes found to robustly associate in both analyses (All four groups and Representative phenotype)				
			All four groups					Representative phenotype									
			Tau			Amyloid-β		Tau			Amyloid-β		Tau			Amyloid-β	
			Braak	CP13	Ab39	Thal	33.1.1	Braak	CP13	Ab39	Thal	33.1.1	Braak	CP13	Ab39	Thal	33.1.1
Step 1: AD Literature	n/a	***PSEN2***															
	n/a	***SIRT1***															
Step 2: Bioinformatic Prioritization	Representative	***ALOX15B***															
	Representative	***ANGPT2***															
	Representative	***CXCL1***															
	Representative	***DNAAF1***															
	Representative	***DNAI1***															
	Extreme	***DYDC2***															
	Representative	***LRRC48***															
	Both	***MAPK15***															
	Representative	***OR7A5***															
	Representative	***PYDC1***															
	Extreme	***RBP1***															
	Representative	***RP11-81K2.1***															
	Representative	***SERPINA5***															
	Representative	***TAC1***															
Step 3: Process Networks	Representative	***ATR***															
	Representative	***BCL2***															
	Extreme	***CAV1***															
	Representative	***CDKN2C***															
	Representative	***CNOT8***															
	Representative	***CTCF***															
	Representative	***CXCL1***															
	Representative	***DAXX***															
	Extreme	***DST***															
	Representative	***EML4***															
	Extreme	***ERBB2IP***															
	Representative	***FEM1B***															
	Representative	***FOXO4***															
	Extreme	***IFITM2***															
	Representative	***INSR***															
	Representative	***IRS2***															
	Representative	***LCOR***															
	Extreme	***MAGED1***															
	Representative	***MAX***															
	Representative	***NCOA1***															
	Representative	***NOS1***															
	Representative	***PPM1D***															
	Representative	***RAD52***															
	Representative	***RBBP7***															
	Representative	***RELA***															
	Representative	***RYBP***															
	Representative	***SIPA1***															
	Representative	***SLC38A2***															
	Representative	***SUN2***															
**Total Genes Associated***			30	0	35	36	0	13	33	35	23	0	13	0	35	23	0

Supplementary Fig. 3 | RNA-Seq gene expression level associations with neuropathologic measures. In this table we provide association studies of our 44 neuropathologically prioritized genes. The goal of Step 4 of Fig. 2 was to evaluate the relationship of the genes identified in Steps 1-3 with neuropathologic measures of tau and amyloid-β. Tau measures included Braak tangle stage, an early marker of tangle maturity (CP13), and an advanced marker of tangle maturity (Ab39). Amyloid-β measures included Thal amyloid phase and a pan-Aβ marker (33.1.1). Linear regression was used to examine association of gene expression levels across all four groups, which included controls, hippocampal sparing AD, typical AD, and limbic predominant AD. To enrich for genes with robust associations, the representative phenotype (controls and typical AD) was additionally evaluated. The final column shows the overlap in association between all four groups and the representative phenotype. The gray boxes indicates that the gene expression level association with neuropathology was identified with a FDR<0.25 and p<0.05. *CXCL1 only considered once in Total Genes Associated row. Acronyms: AD=Alzheimer's Disease, false discovery rate=FDR.

Characteristic	Controls (n=32)	AD neuropathologic subtypes (n=150)			AD specific p-value
		HpSp AD (n=36)	Typical AD (n=79)	Limbic predominant AD (n=35)	
Males (% total of AD type)	15/32 (47%)	14/36 (39%)	26/79 (33%)	16/35 (46%)	0.42
APOE ε4, %	5/32 (16%)	14/36 (39%)	40/79 (51%)	23/35 (66%)	0.076
**Clinical findings**					
Age at onset, yr.	NA (NA, NA)	66 (60, 72)	73 (67, 79)	79 (72, 82)	<0.001
Disease duration, yr.	NA (NA, NA)	8.4 (6.6, 9.3)	8.3 (6.3, 10)	9.4 (6.9, 11)	0.36
Final MMSE, points/yr.	29 (27, 29)	8 (1.5, 12)	14 (9.3, 22)	16 (8, 22)	0.15
**Postmortem findings**					
Age at death, yr.	87 (80, 91)	72 (68, 80)	82 (74, 88)	87 (82, 93)	<0.001
Braak tangle stage	II (II, III)	VI (V, VI)	VI (V, VI)	VI (V, VI)	0.26
Thal amyloid phase	0 (0, 2)	5 (5, 5)	5 (5, 5)	5 (4, 5)	0.097
Early tangle marker (CP13), %	0.4 (0.1, 1.7)	11 (7.6, 16)	15 (11, 18)	15 (11, 19)	0.12
Mature tangle marker (Ab39), %	0.06 (0.04, 0.09)	0.24 (0.11, 0.43)	0.33 (0.21, 0.64)	0.54 (0.39, 0.77)	<0.001
Pan-Aβ marker (33.1.1), %	0.12 (0.07, 0.21)	0.38 (0.24, 0.62)	0.41 (0.31, 0.59)	0.49 (0.32, 0.64)	0.58
Astrogliosis marker (GFAP), %	31 (23, 37)	33 (23, 40)	43 (34, 52)	53 (43, 59)	<0.001
Activated microglial marker (CD68), %	0.13 (0.08, 0.21)	0.23 (0.17, 0.34)	0.30 (0.22, 0.41)	0.38 (0.30, 0.54)	0.0020
Endothelial cell marker (CD34), %	0.73 (0.58, 0.83)	0.47 (0.37, 0.78)	0.67 (0.49, 0.97)	0.90 (0.67, 0.99)	<0.001

Supplementary Fig. 4 | NanoString cohort characteristics. Table contains demographic, clinical, and neuropathologic characteristics of the NanoString cohort. Data are presented as: sample size (percentage) or median (25th percentile, 75th percentile). Digital pathology measures are presented as percentage burden of immunopositive staining. Acronyms: AD=Alzheimer's disease, HpSp=hippocampal sparing AD, MMSE=Mini Mental State Examination, n=sample size, yr=years. Note: Normal controls were not included in two-sided Kruskal Wallis Rank Sum Test, thus p-values specifically reflect group-wise comparisons among AD subtypes.

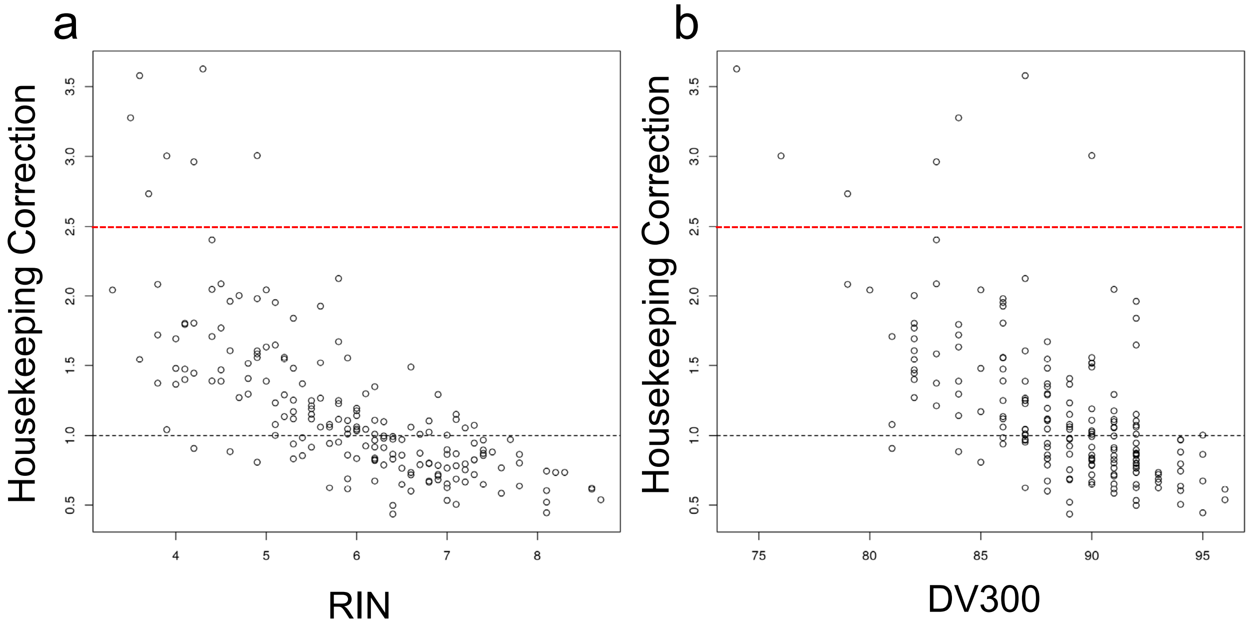

Supplementary Fig. 5 | Exclusion criteria for normalized NanoString data. Raw gene counts for 44 genes quantified by NanoString were normalized using a housekeeping correction factor. To visualize the RNA quality control measures of the seven excluded samples shown to have a greater than 2.5 fold adjustment a, RIN and b, DV300 were plotted against the housekeeping correction factor. Housekeeping genes included: *C1orf43, CHMP2A, EMC7, GAPDH, GPI, PSMB2, PSMB4, RAB7A, REEP5, SNRPD3, VCP, VPS29*. Acronyms: DV300=distribution value over 300 base pairs, RIN=RNA integrity number.

Gene	Pearson Correlation	R-squared	p-value
SERPINA5	0.899	0.81	<0.001
RYBP	0.83	0.69	<0.001
SLC38A2	0.99	0.97	<0.001
FEM1B	0.83	0.68	<0.001
PYDC1	0.74	0.55	<0.001

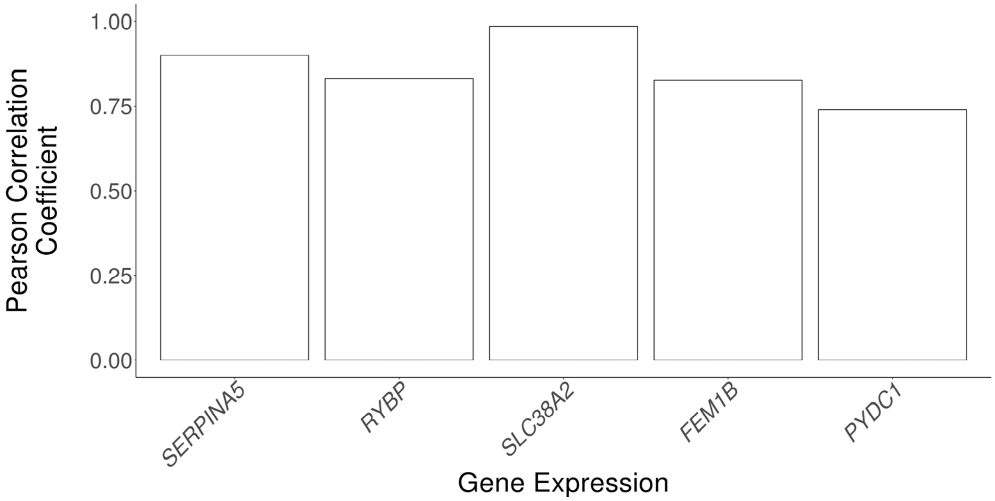

Supplementary Fig. 6 | RNA-Seq and NanoString gene expression levels strongly correlate. Gene expression levels measured using RNA-Seq and NanoString platforms were strongly correlated using two-sided Pearson correlation analysis.

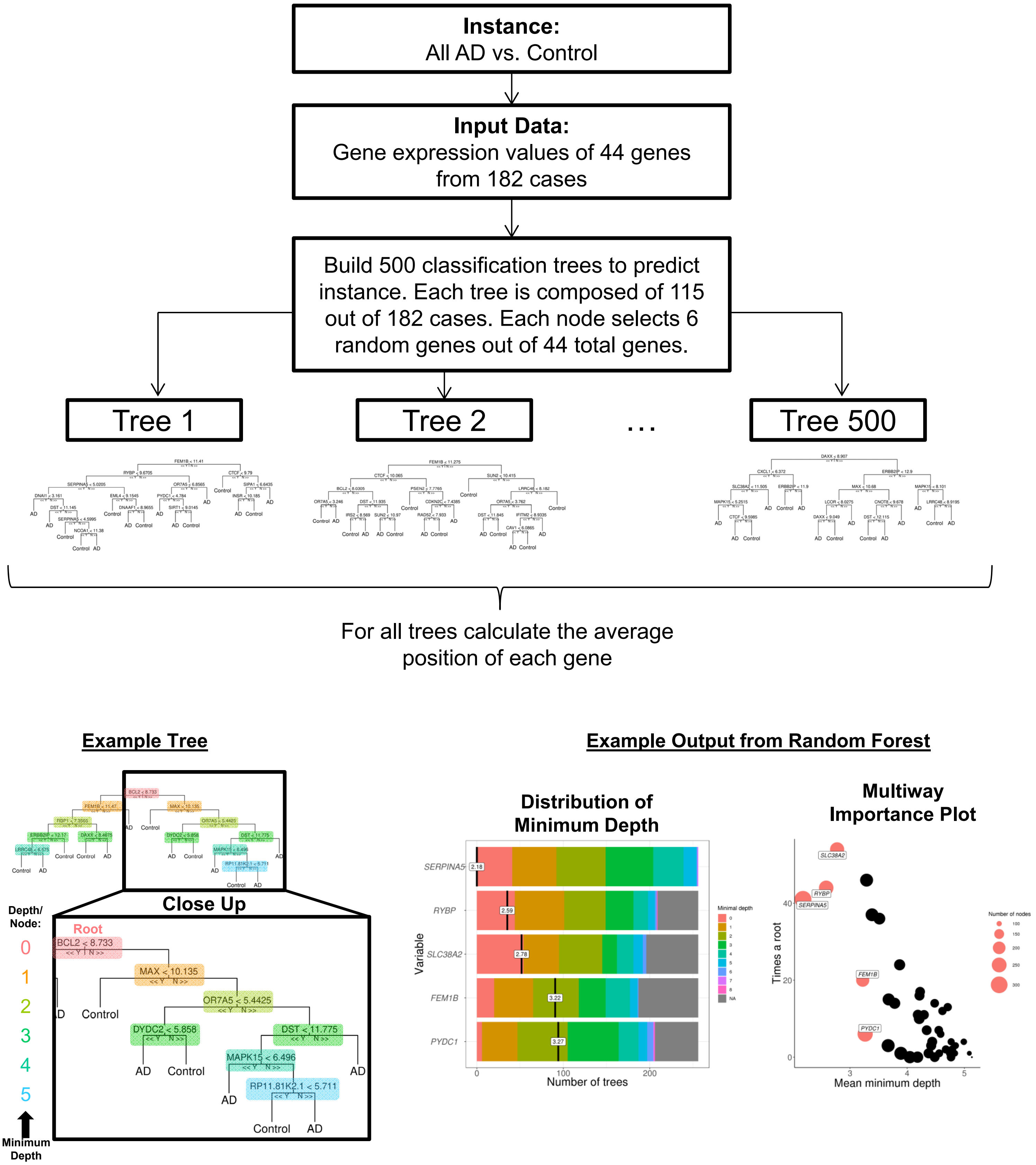

Supplementary Fig. 7 | Figure legend carried over to the next page

Supplementary Fig. 7 | Visual example of Random Forest Random forest analysis workflow and inputs. The goal of performing an in silico experiment using the random forest algorithm is to predict an instance. We performed one random forest analysis to examine the representative phenotype, another to examine the extreme phenotype, and a final one to examine all AD cases (including hippocampal sparing AD, typical AD and limbic predominant AD) versus control. Herein we provide an example of the latter, which corresponds to Fig. 4. We utilized the NanoString gene expression data that contained normalized gene expression values of 44 genes from 182 hippocampi (150 AD cases and 32 controls). Our algorithm built 500 classification trees each composed of a different random sample of 63% (116/182) of our total number of cases. At each node, 6 of the 44 genes were randomly selected and the best predictor was incorporated into the tree. The higher the gene is to the top in a decision tree, the more predictive it is of AD. The average gene position was calculated and can be represented on a minimum depth plot or a multiway importance plot. Acronyms: AD=Alzheimer’s Disease.

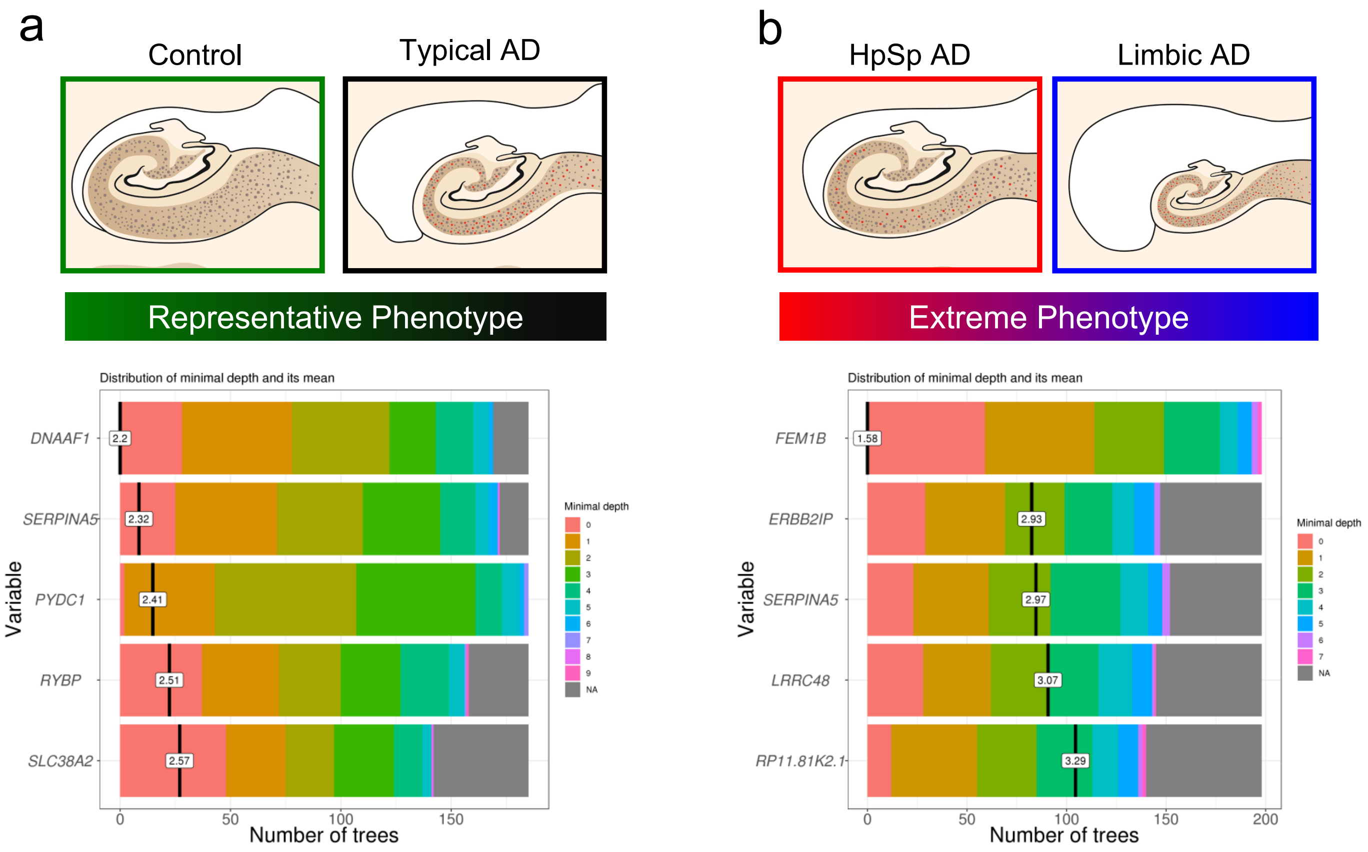

Supplementary Fig. 8 | Random Forest algorithm applied to extreme and representative phenotype. a, Random forest algorithm applied to the representative phenotype (control versus typical AD) identified DNAAF1 as the most predictive gene of typical AD versus control followed by *SERPINA5, PYDC1, RYBP and SLC38A2*. b, Random forest algorithm applied to the extreme phenotype (hippocampal sparing AD versus limbic predominant AD) revealed *FEM1B* to be the most predictive of limbic predominant AD when compared to hippocampal sparing AD. Other predictive genes included *ERBB2IP, SERPINA5, LRRC48 and RP11.81K2.1*. Acronyms: AD=Alzheimer's Disease, HpSp=hippocampal sparing, Limbic=limbic predominant. Note: Variable importance summaries used to prioritize are found in Supplementary Results 1.

**Variable (unit change)**	**OR (95% CI)**	**p-value**
Sex (male)	0.67 (0.24, 1.9)	0.45
Age (15 years)	0.57 (0.23, 1.4)	0.23
SERPINA5 gene expression (2.0)	2.3 (1.3, 4.2)	0.0040
SLC38A2 gene expression (1.2)	7.6 (1.5, 39)	0.016
FEM1B gene expression (0.38)	2.4 (0.82, 7.4)	0.11
PYDC1 gene expression (1.4)	0.49 (0.25, 0.97)	0.040
RYBP gene expression (0.51)	0.77 (0.26, 2.3)	0.65

Supplementary Fig. 9 | Association between nomogram variables and likelihood of Alzheimer's Disease. Multiple logistic regression models were used to quantify the degree to which the variables discriminated AD from control. The table depicts each variable from the nomogram with its corresponding OR and 95% CI. OR is a statistic that quantifies the strength of association between two events, which in this case are likelihood of neuropathologically diagnosed AD. An odds ratio greater than 1 indicates the outcome is more likely whereas an odds ratio less than 1 indicates the outcome is less likely. For example, for every 2-unit increase in gene expression value of *SERPINA5* in the hippocampus the odds of being neuropathologically diagnosed as AD in the NanoString cohort increases two-fold (OR=2., 95% CI: 1.3, - 4.2; p=0.004). Acronyms: OR=odds ratio, CI=95% confidence interval.

a **How to use a nomogram**

1. Plot sex, age and gene expression values (■)
2. Add up total points (↑)
3. Plot total points (■)
4. Obtain predicted value (↓■)

b

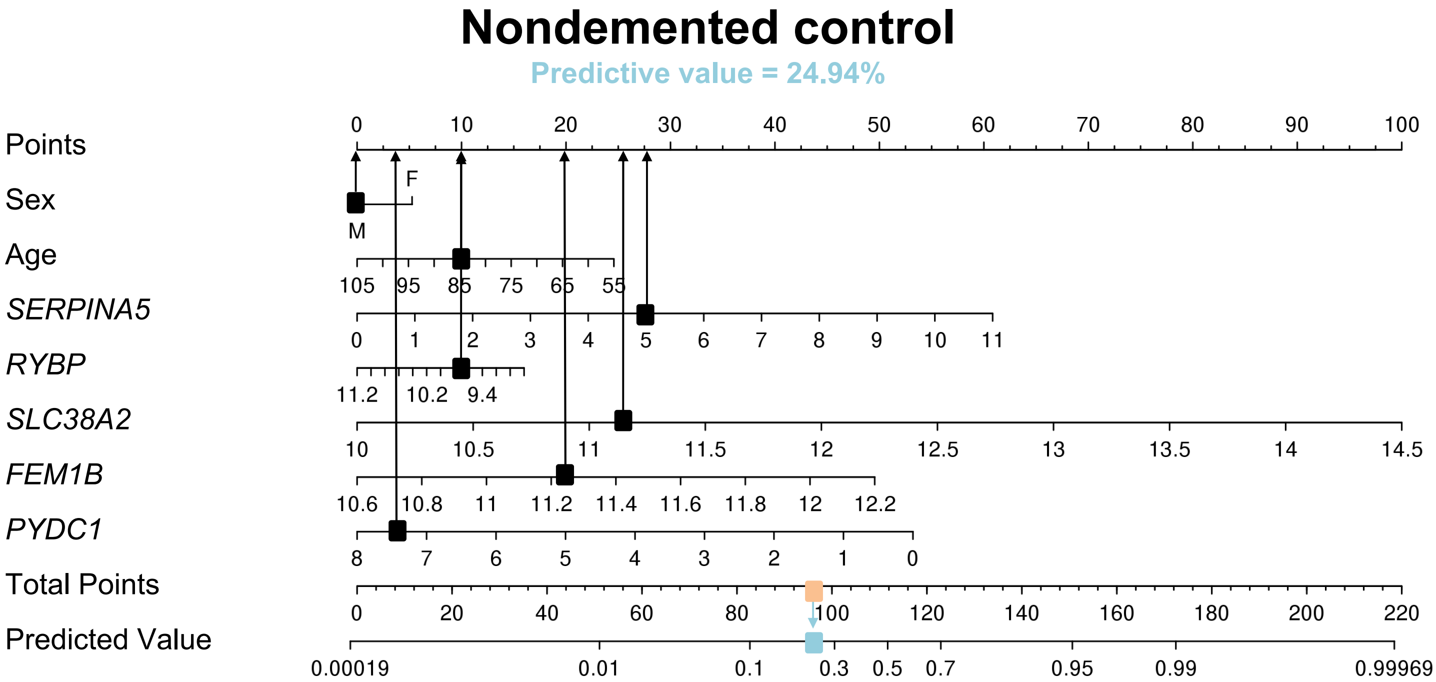

c

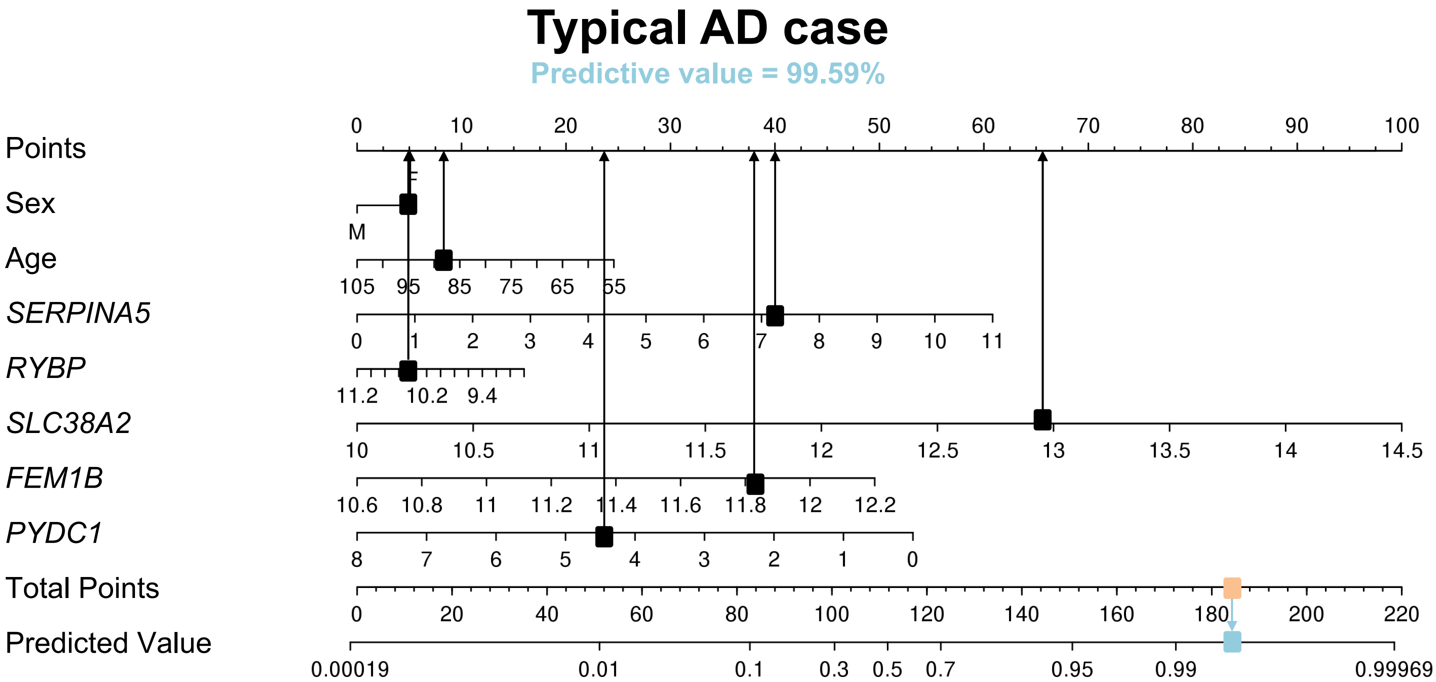

Supplementary Fig. 10 | Figure legend carried over to the next page

Supplementary Fig. 10 | Example of Nomogram utility. a, Overview on how to use a nomogram to obtain predicted value for an individual sample b, An 84 year old (10 pts) male (0 pts) was found to have minimal AD pathology upon neuropathologic inspection (Braak=0 and Thal=0). Normalized hippocampal gene expression from NanoString analyses of the top 5 genes identified levels of *SERPINA5* at 4.95 (27 pts), *RYBP* at 9.66 (10 pts), *SLC38A2* at 11.2 (26 pts), *FEM1B* at 11.2 (20 pts), and *PYDC1* at 7.42 (4 pts). His 97 total points translate to a low predicted probability (24.9%) of being neuropathologically diagnosed AD. c, An 88 year old (8 pts) female (5 pts) was found to have significant AD pathology (Braak=VI and Thal=5) upon neuropathologic inspection. Normalized hippocampal gene expression from NanoString analyses of the top 5 genes identified levels of *SERPINA5* at 7.20 (40 pts), *RYBP* at 10.5 (5 pts), *SLC38A2* at 12.9 (65 pts), *FEM1B* at 11.8 (37 pts), and *PYDC1* at 4.52 (23 pts). Her 184 total points translate to a high predicted probability (99.6%) of being neuropathologically diagnosed AD. Acronyms: AD=Alzheimer's Disease, pt=points. Note: These statistics correspond to Fig. 4d of the main manuscript.

Markers and covariates	*SERPINA5*		*RYBP*		*SLC38A2*		*FEM1B*		*PYDC1*	
	**Coeff.**	**P-value**	**Coeff.**	**P-value**	**Coeff.**	**P-value**	**Coeff.**	**P-value**	**Coeff.**	**P-value**
Early tangle	0.002	0.62	0.014	0.14	**0.040**	**0.011**	**0.076**	**0.000**	**0.030**	**0.028**
Advanced tangle	**0.041**	**0.010**	0.015	0.13	**0.031**	**0.026**	0.00	0.88	0.007	0.29
Pan-Aβ	0.002	0.58	0.002	0.63	0.001	0.74	0.016	0.11	0.000	0.99
Microglial	0.020	0.075	0.024	0.052	0.024	0.052	0.019	0.087	0.000	0.88
Endothelial	0.014	0.14	0.017	0.097	0.012	0.17	0.008	0.25	0.000	0.79
Astroglial	0.004	0.40	0.002	0.60	0.002	0.58	0.039	0.013	0.000	0.84
Age at death	0.004	0.40	**0.082**	**0.00**	0.022	0.060	**0.054**	**0.003**	0.024	0.052
Sex (Male)	0.014	0.13	0.00	0.90	0.00	0.88	0.001	0.67	0.004	0.44
*APOE ε*4 status	0.00	0.99	0.017	0.098	0.017	0.10	0.001	0.74	0.000	0.81

Supplementary Fig. 11 | Linear regression modeling of gene expression and digital pathology findings. a, Table with each of the top 5 genes and its association with markers of early tangle maturity (CP13), advanced tangle maturity (Ab39), pan-Aβ (33.1.1), microglia (CD68), endothelia (CD34), astroglia (GFAP). Covariates included age at death, sex (male), and APOE ε4 status modeled together with markers. All statistically significant values (i.e. $p<0.05$) are bolded. Note: These statistics correspond to Fig. 4h of the main manuscript. Coeff=coefficient of partial determination. Bold denotes significant values.

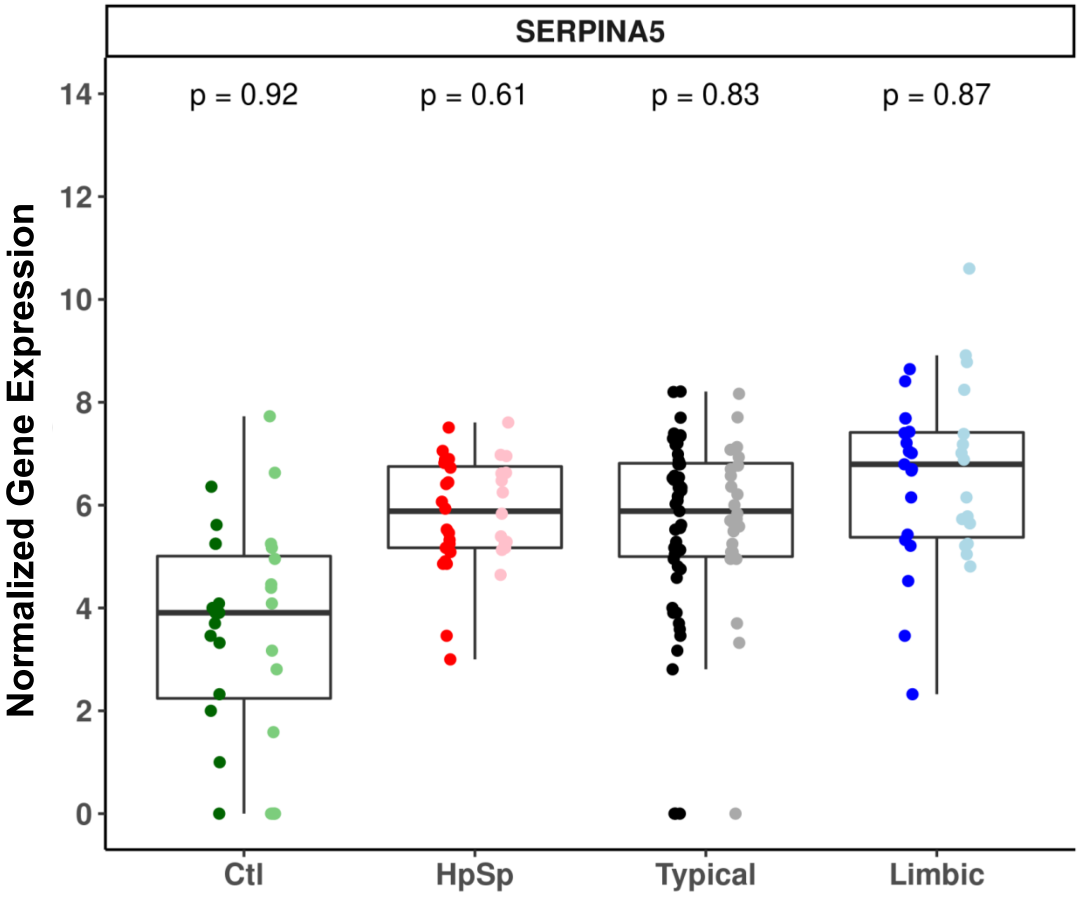

Supplementary Fig. 12 | *SERPINA5* gene expression stratified by sex. Statistical analyses from two-sided Wilcoxon Rank Sum tests showing pair-wise comparisons of *SERPINA5* gene expression levels in the NanoString cohort did not differ when males and females were compared. Sex differences were stratified within controls, hippocampal sparing AD, typical AD, and limbic predominant AD. Each jitter plot overlay displays females on the left (darker color) and males on the right (lighter color). Note: Box plots in are derived from n=182 independent samples. Box plots are displayed at the 25th and 75th percentile with the median line. Whiskers are drawn to the largest and smallest values that are within 1.5 times the interquartile range from the upper or lower quartile. For observations larger or smaller than this distance, they are shown as observations outside the whiskers.

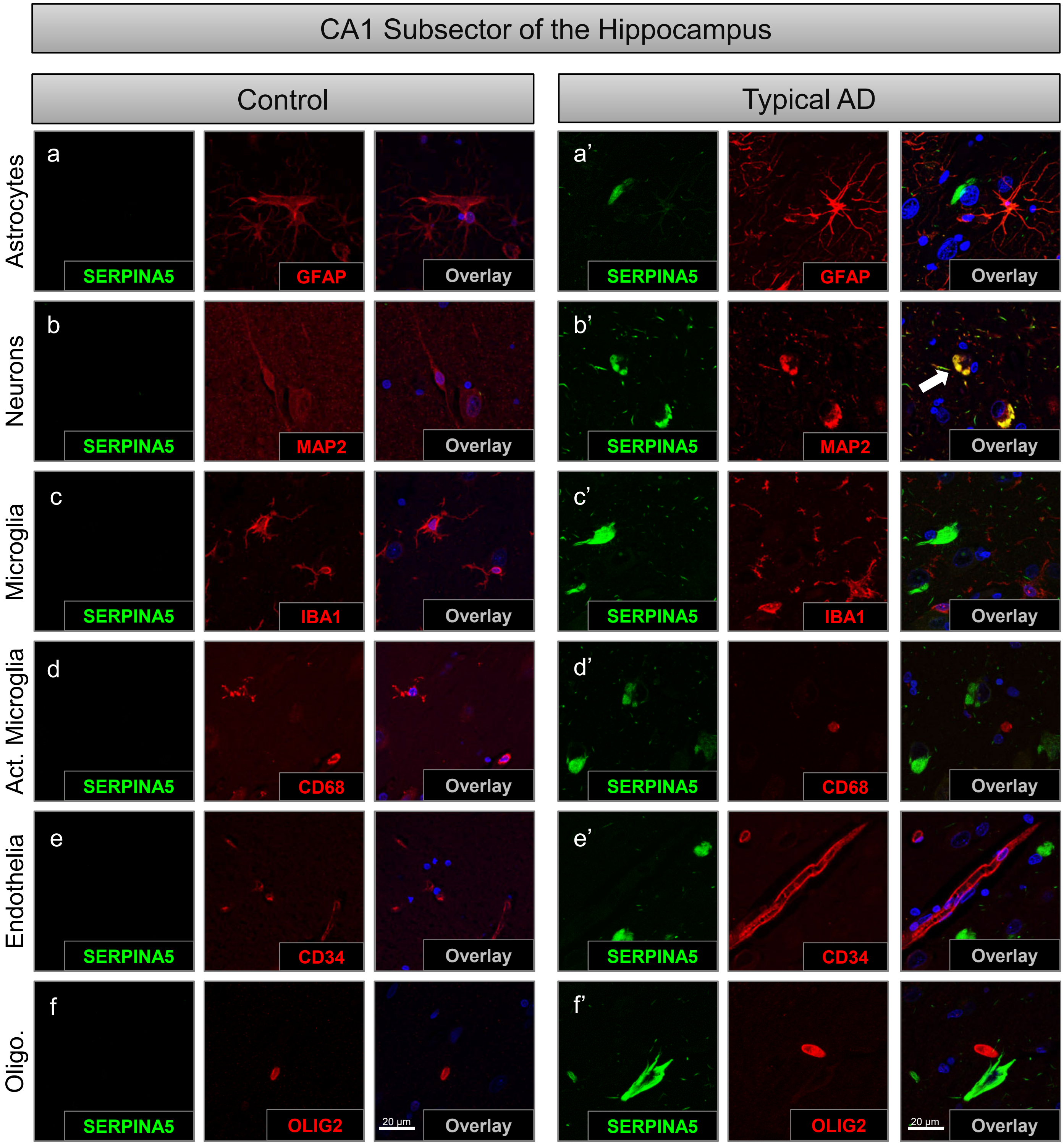

Supplementary Fig. 13 | SERPINA5 protein expression in brain cell types. The CA1 subsector of the posterior hippocampus was investigated using co-immunofluorescence to evaluate cell type specificity of SERPINA5: a-a’, GFAP (astrocytes), b-b’, MAP2 (neurons), c-c’, IBA1 (microglia), d-d’, CD68 (activated microglia), e-e’ CD34 (endothelia), f-f’, OLIG2 (oligodendrocytes). a-f, SERPINA5 protein was not found in any cell type of a 60 year old male control (Braak=0, Thal=1). a’-f’, SERPINA5 protein expression in an 86 year old female AD case (Braak=VI, and Thal=5) was found in neurofibrillary tangle-bearing neurons (arrow, b’). a-f’, Overlay includes SERPINA5 (green), cell-specific stain (red), and DAPI (blue). Immunofluorescent staining experiments were performed successfully in triplicate for AD and controls. Scale bar represents 20 µm. Acronyms: Act=Activated, AD=Alzheimer’s Disease, Oligo=oligodendrocytes.

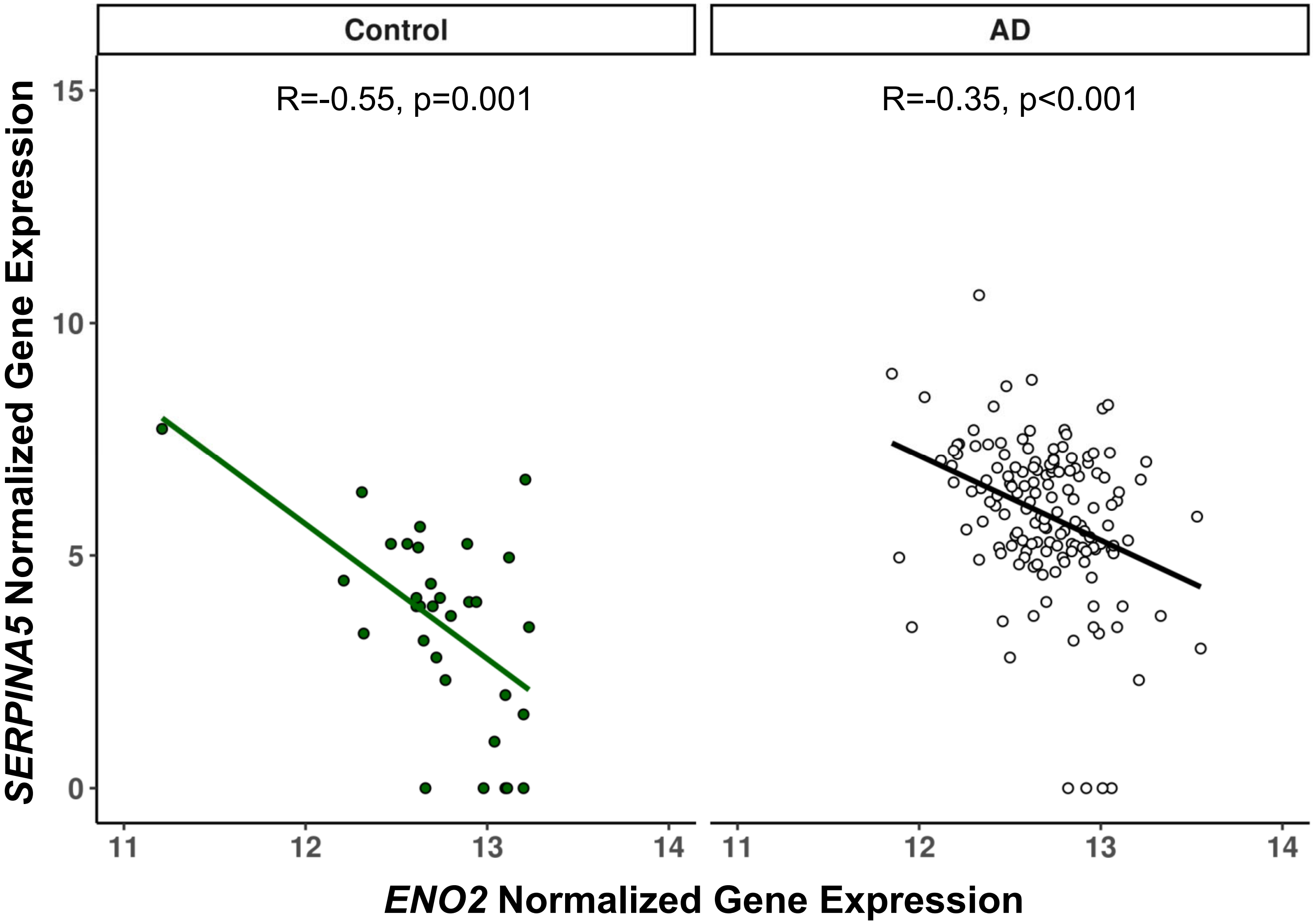

Supplementary Fig. 14 | *SERPINA5* gene expression correlates with neuronal marker *ENO2*. Gene expression levels from the neuronal marker *ENO2* associated with SERPINA5 levels in both controls (R=-0.55, p=0.001) and AD cases (R=-0.35, p<0.001) from the NanoString cohort using two-sided Pearson correlation analysis.

Characteristic	Controls (n=10)	AD neuropathologic subtypes (n=40)			AD specific p-value
		HpSp AD (n=20)	Typical AD (n=20)	Limbic predominant AD (n=20)	
Males (% total of AD type)	5/10 (50%)	15/20 (75%)	11/20 (55%)	3/20 (15%)	< 0.001
APOE ε4*, %	0/1 (0%)	10/17 (59%)	13/19 (68%)	6/9 (67%)	0.83
**Clinical findings**					
Age at onset, yr.	NA (NA, NA)	65 (58, 68)	72 (65, 76)	81 (78, 87)	< 0.001
Disease duration, yr.	NA (NA, NA)	7.9 (6.3, 10)	10 (7.3, 15)	6.8 (6, 8.6)	0.18
**Postmortem findings**					
Age at death, yr.	76 (60, 81)	71 (68, 74)	82 (76, 85)	87 (84, 91)	< 0.001
Braak tangle stage	I (I, II)	VI (V, VI)	VI (V, VI)	V (IV,VI)	0.011
Thal amyloid phase	1 (0, 2)	5 (5, 5)	5 (5, 5)	5 (5, 5)	0.87
Average hippocampal SERPINA5, %	0.010 (0.010, 0.020)	2.6 (1.8, 3.9)	4.0 (2.4, 8.4)	5.3 (2.0, 9.8)	0.011
CA1, %	0.010 (0.010, 0.020)	1.0 (0.77, 1.4)	2.0 (0.94, 2.8)	1.8 (0.78, 3.8)	0.015
Subiculum, %	0.020 (0.010, 0.020)	1.6 (0.76, 2.4)	2.2 (1.4, 4.9)	3.5 (1.2, 5.6)	0.014
Average cortical SERPINA5, %	0.010 (0.010, 0.020)	5.8 (3.9, 7.6)	5.4 (1.4, 7.6)	1.7 (0.95, 2.8)	<0.001
Superior temporal, %	0.010 (0.010, 0.020)	1.2 (0.85, 1.7)	1.4 (0.44, 2.8)	0.71 (0.43, 1.1)	0.075
Inferior parietal, %	0.010 (0.010, 0.020)	1.8 (1.2, 2.6)	1.4 (0.54, 3.3)	0.44 (0.23, 0.67)	<0.001
Mid-frontal, %	0.010 (0.010, 0.020)	2.2 (1.6, 3.2)	1.8 (0.53, 2.2)	0.19 (0.12, 0.43)	<0.001

Supplementary Fig. 15 | Quantitative immunohistochemical burden of SERPINA5 measured by digital pathology. Demographic and clinical characteristics are provided for the 10 controls and 60 AD cases independently examined. The hippocampal SERPINA5 burden (averaged CA1 and subiculum) increased in a general monotonic direction among AD subtypes. In contrast, cortical SERPINA5 burden (averaged superior temporal, inferior parietal, mid-frontal) decreases in a general monotonic direction among AD subtypes. Data are presented as: median (25th percentile, 75th percentile). Digital pathology measures are presented as percentage burden of immunopositive staining. Acronyms: AD=Alzheimer's disease, HpSp=hippocampal sparing AD. Note: Normal controls were not included in two-sided Kruskal Wallis Rank Sum Test, thus p-values specifically reflect group-wise comparisons among AD subtypes. *Denominators for *APOE* ε4 reflect total number with frozen tissue available for genotyping.

AD Subtype	Total reads	Used reads	Mapped reads	Mapped reads (Genome)	Mapped reads (Junction)	Gene count	Exon count
Ctl-1	107,651,934	107,568,111	93,194,746 (86.6)	83,514,090 (77.6)	9,680,656 (9.0)	33,353,018 (31.0)	173,910,437 (161.5)
Ctl-2	143,957,388	143,812,328	123,167,238 (85.6)	111,860,651 (77.7)	11,306,587 (7.9)	43,690,722 (30.3)	236,111,216 (164.0)
Ctl-3	179,380,730	179,357,440	147,567,698 (82.3)	129,896,840 (72.4)	17,670,858 (9.9)	61,174,456 (34.1)	330,677,695 (184.3)
Ctl-4	118,329,800	118,199,155	98,321,008 (83.1)	86,910,578 (73.4)	11,410,430 (9.6)	38,537,231 (32.6)	209,250,869 (176.8)
Ctl-5	134,746,118	134,617,790	116,033,251 (86.1)	98,736,415 (73.3)	17,296,836 (12.8)	53,015,332 (39.3)	293,307,193 (217.7)
Ctl-6	118,919,334	118,857,614	103,343,443 (86.9)	89,839,574 (75.5)	13,503,869 (11.4)	43,966,262 (37.0)	240,414,294 (202.2)
Ctl-7	102,808,398	102,728,580	88,127,185 (85.7)	76,617,237 (74.5)	11,509,948 (11.2)	37,153,641 (36.1)	209,289,438 (203.6)
Ctl-8	153,409,250	153,254,804	128,699,480 (83.9)	114,521,058 (74.7)	14,178,422 (9.2)	48,480,606 (31.6)	264,765,534 (172.6)
Ctl-9	112,510,692	112,408,836	93,514,202 (83.1)	83,352,847 (74.1)	10,161,355 (9.0)	37,432,246 (33.3)	209,566,548 (186.3)
Ctl-10	102,883,862	102,801,693	86,186,616 (83.8)	76,974,145 (74.8)	9,212,471 (9.0)	34,656,660 (33.7)	188,725,481 (183.4)
Ctl-11	138,409,450	138,268,143	114,903,154 (83.0)	104,115,662 (75.2)	10,787,492 (7.8)	42,052,850 (30.4)	224,181,633 (162.0)
Ctl-12	137,351,268	137,208,841	117,197,326 (85.3)	105,333,157 (76.7)	11,864,169 (8.6)	42,096,302 (30.6)	230,046,259 (167.5)
Ctl-13	125,903,118	125,780,042	107,610,184 (85.5)	90,092,659 (71.6)	17,517,525 (13.9)	54,993,809 (43.7)	311,179,897 (247.2)
Ctl-14	165,767,984	165,747,538	140,771,037 (84.9)	123,432,263 (74.5)	17,338,774 (10.5)	57,999,533 (35.0)	319,470,580 (192.7)
Ctl-15	127,584,888	127,458,173	108,436,878 (85.0)	94,494,689 (74.1)	13,942,189 (10.9)	45,757,687 (35.9)	252,590,533 (198.0)
HpSp-1	125,276,906	125,170,419	106,938,562 (85.4)	98,670,718 (78.8)	8,267,844 (6.6)	31,258,536 (25.0)	162,297,018 (129.6)
HpSp-2	112,391,398	112,298,101	96,120,757 (85.5)	87,315,274 (77.7)	8,805,483 (7.8)	34,170,760 (30.4)	182,357,957 (162.3)
HpSp-3	124,563,828	124,462,427	106,689,763 (85.7)	94,358,412 (75.8)	12,331,351 (9.9)	44,222,969 (35.5)	237,704,396 (190.8)
HpSp-4	257,886,536	257,843,755	223,556,514 (86.7)	197,410,263 (76.5)	26,146,251 (10.1)	97,530,807 (37.8)	548,977,311 (212.9)
HpSp-5	240,891,618	240,837,051	204,623,466 (84.9)	180,740,549 (75.0)	23,882,917 (9.9)	88,254,170 (36.6)	479,412,236 (199.0)
HpSp-6	108,879,028	108,788,404	92,934,112 (85.4)	79,679,966 (73.2)	13,254,146 (12.2)	39,141,681 (35.9)	214,916,975 (197.4)
HpSp-7	135,137,536	135,003,369	115,152,555 (85.2)	101,068,299 (74.8)	14,084,256 (10.4)	45,468,489 (33.6)	239,973,913 (177.6)
HpSp-8	102,379,238	102,298,930	89,748,321 (87.7)	80,618,042 (78.7)	9,130,279 (8.9)	35,746,929 (34.9)	191,239,060 (186.8)
HpSp-9	103,100,460	103,016,548	90,481,939 (87.8)	81,212,317 (78.8)	9,269,622 (9.0)	33,389,408 (32.4)	177,644,705 (172.3)
HpSp-10	111,566,254	111,459,962	97,426,140 (87.3)	85,344,209 (76.5)	12,081,931 (10.8)	41,897,472 (37.6)	229,765,923 (205.9)
Typical-1	97,194,370	97,119,394	83,681,667 (86.1)	73,699,151 (75.8)	9,982,516 (10.3)	36,072,349 (37.1)	193,412,105 (199.0)
Typical-2	340,499,070	340,422,280	284,816,399 (83.6)	248,762,075 (73.1)	36,054,324 (10.6)	119,359,928 (35.1)	633,471,756 (186.0)
Typical-3	84,807,392	84,735,639	72,524,774 (85.5)	64,581,000 (76.2)	7,943,774 (9.4)	30,375,552 (35.8)	165,606,914 (195.3)
Typical-4	118,105,530	117,990,305	101,555,777 (86.0)	91,640,778 (77.6)	9,914,999 (8.4)	39,511,999 (33.5)	205,378,512 (173.9)
Typical-5	146,395,500	146,270,388	124,619,457 (85.1)	113,148,447 (77.3)	11,471,010 (7.8)	45,972,822 (31.4)	241,880,059 (165.2)
Typical-6	127,957,452	127,826,202	107,764,096 (84.2)	95,164,934 (74.4)	12,599,162 (9.8)	40,650,935 (31.8)	219,897,728 (171.9)
Typical-7	140,652,042	140,510,428	118,797,157 (84.5)	103,874,665 (73.9)	14,922,492 (10.6)	51,881,008 (36.9)	280,995,043 (199.8)
Typical-8	169,673,950	169,651,345	135,795,507 (80.0)	121,626,439 (71.7)	14,169,068 (8.4)	53,375,589 (31.5)	285,082,193 (168.0)
Typical-9	261,480,608	261,434,148	226,628,485 (86.7)	199,107,889 (76.1)	27,520,596 (10.5)	99,517,980 (38.1)	553,777,854 (211.8)
Typical-10	148,154,652	148,017,561	124,021,870 (83.7)	112,545,054 (76.0)	11,476,816 (7.7)	42,183,333 (28.5)	220,340,383 (148.7)
Typical-11	112,105,860	112,016,935	94,369,236 (84.2)	84,816,813 (75.7)	9,552,423 (8.5)	36,967,227 (33.0)	198,202,069 (176.8)
Typical-12	104,536,288	104,450,587	89,427,140 (85.5)	80,053,842 (76.6)	9,373,298 (9.0)	32,423,808 (31.0)	173,988,951 (166.4)
Typical-13	112,087,790	111,979,772	96,506,791 (86.1)	85,997,307 (76.7)	10,509,484 (9.4)	37,243,917 (33.2)	203,086,222 (181.2)
Typical-14	154,244,518	154,165,681	130,499,592 (84.6)	114,971,350 (74.5)	15,528,242 (10.1)	55,799,738 (36.2)	308,004,109 (199.7)
Typical-15	113,086,410	112,994,111	93,418,875 (82.6)	83,383,790 (73.7)	10,035,085 (8.9)	34,804,902 (30.8)	181,597,976 (160.6)
Typical-16	119,138,942	119,073,285	101,498,158 (85.2)	90,320,377 (75.8)	11,177,781 (9.4)	40,819,281 (34.3)	222,431,407 (186.7)
Typical-17	119,760,200	119,661,599	100,836,962 (84.2)	90,348,949 (75.4)	10,488,013 (8.8)	36,106,863 (30.1)	190,751,545 (159.3)
Typical-18	111,241,066	111,139,740	96,584,111 (86.8)	84,083,649 (75.6)	12,500,462 (11.2)	44,159,779 (39.7)	241,863,635 (217.4)
Typical-19	130,626,166	130,517,303	110,029,814 (84.2)	98,256,799 (75.2)	11,773,015 (9.0)	44,423,365 (34.0)	252,761,773 (193.5)
Typical-20	134,250,278	134,146,164	113,882,842 (84.8)	101,889,020 (75.9)	11,993,822 (8.9)	41,785,216 (31.1)	222,678,517 (165.9)
Limbic-1	128,055,138	127,935,605	109,770,323 (85.7)	98,593,301 (77.0)	11,177,022 (8.7)	41,791,357 (32.6)	227,169,478 (177.4)
Limbic-2	131,223,754	131,097,454	110,770,095 (84.4)	98,100,780 (74.8)	12,669,315 (9.7)	40,918,851 (31.2)	228,508,418 (174.1)
Limbic-3	148,506,328	148,345,297	125,386,916 (84.4)	111,145,772 (74.8)	14,241,144 (9.6)	47,458,664 (32.0)	250,901,773 (169.0)
Limbic-4	122,503,752	122,408,327	105,169,514 (85.9)	93,676,424 (76.5)	11,493,090 (9.4)	41,497,611 (33.9)	224,512,856 (183.3)
Limbic-5	137,500,040	137,380,927	116,574,780 (84.8)	107,220,033 (78.0)	9,354,747 (6.8)	36,927,991 (26.9)	193,093,063 (140.4)
Limbic-6	113,330,370	113,226,143	96,216,328 (84.9)	86,551,935 (76.4)	9,664,393 (8.5)	36,998,748 (32.6)	198,689,386 (175.3)
Limbic-7	103,100,544	103,021,768	88,905,382 (86.2)	78,562,694 (76.2)	10,342,688 (10.0)	36,865,089 (35.8)	200,719,088 (194.7)
Limbic-8	143,963,148	143,805,475	121,620,275 (84.5)	110,174,778 (76.5)	11,445,497 (8.0)	44,440,332 (30.9)	242,905,288 (168.7)
Limbic-9	121,590,604	121,472,061	106,849,088 (87.9)	93,720,771 (77.1)	13,128,317 (10.8)	44,069,682 (36.2)	236,142,675 (194.2)
Limbic-10	137,338,192	137,195,789	115,045,891 (83.8)	103,861,577 (75.6)	11,184,314 (8.1)	38,278,525 (27.9)	202,203,461 (147.2)

Supplementary Fig. 16 | RNA-Seq raw read counts. Table showing total reads, used reads, mapped reads (genome and junctional), gene count and exon count from RNA-Seq data for all 55 cases sequenced. Number in parenthesis is the percentage of total reads. Ctl=control, HpSp=hippocampal sparing AD, limbic=limbic predominant AD.

a

Dataset	Data Type	Description	SynapseID	DoD
Mayo RNAseq TCX	Expression	Consensus processed RNASeq raw counts	syn8690799	10/2/2019
Mayo RNAseq TCX	Metadata	Individual human and RNAseq	syn3817650	n/a
Mayo RNAseq TCX	Metadata	Quality Control	syn6126114	n/a
MSBB	Expression	Consensus processed RNASeq raw counts	syn8691099	10/2/2019
MSBB	Metadata	Individual human	syn6101474	11/22/2019
MSBB	Metadata	Assay RNAseq	syn6100548	10/2/2019

b

**Dataset**	**Mayo-TCX[h]**	**MSBB-BM22[i]**	**MSBB-BM36[i]**
**Brain Region sampled**	Temporal cortex	Superior temporal gyrus	Parahippocampal gyrus
**Unique sample IDs[a]**	278	264	267
**Gene counts missing[b]**	0	5	4
**Sex check[c]**	2	0	0
**RIN <5[d]**	0	38	48
**PCA outlier[e]**	2	0	0
**Flagged[f]**	15	33	48
**Ethnoracial status[g]**	0	39	40
**Retained**	259	149	127
**AD**	**80**	**70**	**56**
**Control**	**68**	**33**	**30**
**Other/unknown[j]**	111	46	41

c

	**Controls**	**AD**	**p-value**
**Mayo-TCX**	n=68	n=80	
Age at death, yrs	86 (78,89)	85 (78,89)	0.93
Females, %	34/68 (50%)	49/80 (61%)	0.17
APOE ε4, %	8/68 (12%)	42/80 (52%)	<0.001
**MSBB-BM22**	n=33	n=70	
Age at death, yrs	84 (79,90)	86 (80,90)	0.53
Females, %	18/33 (54%)	49/70 (70%)	0.12
APOE ε4, %	2/19 (10%)	16/44 (36%)	0.037
**MSBB-BM36**	n=30	n=56	
Age at death, yrs	85 (75,90)	89 (84,90)	0.16
Females, %	14/30 (47%)	41/56 (73%)	0.015
APOE ε4, %	2/20 (10%)	9/33 (27%)	0.13

Supplementary Fig. 17 | Figure legend carried over to the next page

Supplementary Fig. 17 | AMP-AD RNA-Seq validation datasets reprocessed from Mayo Clinic and Mount Sinai brain bank. a, Dataset names correspond to those referenced throughout the manuscript. DoD is provided, except where data was generated by study authors (NET) and shared within the AMP-AD knowledge portal. SynapseIDs can be searched directly within the portal only (https://adknowledgeportal.synapse.org/). b, The AMP-AD knowledge portal was accessed to download raw gene count and associated metadata files, which was subsequently inspected and underwent quality control. [a]The total number of unique samples IDs that contained the gene count file downloads for a given brain region and dataset prior to exclusions. Sample IDs were excluded [b]if RNA-Seq gene counts had inconsistent values or did not have an associated metadata file; [c]if sex was inconsistent between inference of Y chromosome gene expression and sex noted in their associated metadata files; [d]if RIN <5; [e]if gene counts (counts per million) from principal components analysis of gene expression identified outliers (PC1 or PC2 >4SD from mean); [f]if recommended by Mayo-TCX parent study (syn6126114), and for MSBB if rRNA >5% or the sample with the lowest number of reads in a unique individual that contained two sets of RNA-Seq data (duplicates), and [g]if ethnoracial status was indicated as other than non-Hispanic white to match the current study. [h]Neuropathologically diagnosed AD samples from the Mayo RNA-Seq dataset had a Braak tangle stage ≥IV and controls had a Braak tangle stage ≤III[25,38]. [i]Neuropathologically diagnosed AD samples from the MSBB RNA-Seq dataset had a Braak tangle stage ≥IV and CERAD neuropathologic category ≥2, whereas controls had a Braak tangle stage ≤III and CERAD neuropathology category ≤1[39]. [j]Any samples lacking metadata needed for analysis, had associated neuropathologic information not matching prescribed diagnostic criteria, or samples that contained a non-AD or non-control neuropathologic diagnosis were excluded from our analyses and classified as “other/unknown”. c, Statistical analyses from two-sided Wilcoxon Rank Sum tests showing pair-wise comparisons of age at death, proportion of females, and frequency of the *APOE* ε4 risk allele for each AMP-AD dataset. Data are presented as: sample size (percentage) or median (25th percentile, 75th percentile). Acronyms: DoD=Date of download, MSBB=Mount Sinai VA Medical Center Brain Bank, n/a=not applicable, RNA-Seq=RNA sequencing, TCX=temporal cortex.

a

Antibody	Supplier	Catalog #	Dilution	Antigen Retrieval	Macro
**CP13**	Peter Davies gift	n/a	1:1000	30 min. steam in dH_20	CD
**CD68**	Dako	M0814	1:1000	30 min. steam in dH_20	CD
**SERPINA5**	R&D	MAB1266	1:100	30 min. steam in dH_20	CD
**Ab39**	Shu-Hui Yen gift	n/a	1:350	30 min. steam in dH_20	PPC
**GFAP**	Biogenex	MU020-UC	1:5000	30 min. steam in dH_20	PPC
**33.1.1**	Pritam Das gift	n/a	1:1000	30 min. in 98% Formic acid followed by 30 min. steam in dH_20	PPC
**CD34**	Abcam	Ab81289	1:25	30 min. steam in dH_20	PPC

b

**Color Deconvolution**			
	CP13	CD68	SERPINA5
**Version**	v1	v1	v9
**Positive Color Channel**	3		
**Markup Image Type**	Intensity Ranges		
**Weak Postive Threshold**	235	235	235
**Medium Postive Threshold**	170	180	185
**Strong Positive Threshold**	170	180	185
**Black Threshold**	0	0	0
**Color (1) - Red Component**	0.554541		
**Color (1) - Green Component**	0.614766		
**Color (1) - Blue Component**	0.560845		
**Color (2) - Red Component**	0.072		
**Color (2) - Green Component**	0.99		
**Color (2) - Blue Component**	0.105		
**Color (3) - Red Component**	0.268		
**Color (3) - Green Component**	0.57		
**Color (3) - Blue Component**	0.776		
**Clear Area Intensity**	240		

**Positive Pixel Count**				
	Ab39	GFAP	33.1.1	CD34
**Version**	v9	v9	v2004-08-11	v2004-08-11
**Hue Value**	0.1	0.1	0.1	0.1
**Hue Width**	0.1	0.1	0.1	0.5
**Color Saturation Threshold**	0.08	0.08	0.04	0.04
**Iwp(High)**	235	235	220	220
**Iwp(Low) = Ip(High)**	235	235	175	175
**Ip(Low) = Isp(High)**	235	235	100	100
**Isp (Low)**	0	0	0	0

Default parameters for all: View Width (1000), View Height (1000), Overlap size (0), Image Zoom (1), Markup Compression Type (Same as processsed image), Compression Quality (30), Classifier Neighborhood (0), Classifier (None), Class list (n/a)

c

Primary Antibody	Supplier	Concentration	Antigen Retrieval
GFAP	ABCAM ab33922	1:800	30 min. steam in dH_20
MAP2	ABCAM ab32454	1:200	30 min. steam in dH_20
IBA1	ABCAM ab178847	1:100	30 min. steam in citrate
CD68	Cell Signaling 76437	1:400	30 min. steam in citrate
CD34	ABCAM ab81289	1:200	30 min. steam in dH_20
OLIG2	ABCAM ab109186	1:100	30 min. steam in citrate
SERPINA5	R&D MAB1266	1:100	30 min. steam in dH_20 or citrate (refer to co-stain)
Tau E1	Leonard Petrucelli gift	1:1000	30 min. steam in dH_20
Tau pS396	Abcam 109390	1:1000	30 min. steam in dH_20
Tau E178	Abcam 32057	1:1000	30 min. steam in citrate
IgG2a	R&D MAB003	1:100	N/A

d

Secondary Antibody or Stain	Supplier	Concentration
AlexaFluor488 Goat α Mouse	Invitrogen A11001	1:500
AlexaFluor568 Goat α Rabbit	Invitrogen A11011	1:500
Thioflavin-S	Sigma T1892	1% solution dissolved in water

Supplementary Fig. 18 | Antibody tables. a, List of antibodies used for digital pathology studies. b, List of antibodies used for immunofluorescence studies. c, List of secondary antibodies and stains used for immunofluorescence studies. Acronyms: CD=color deconvolution, dH20=deionized water, Iwp=Intensity weak-positive pixels, Ip=Intensity positive pixels, Isp=Intensity strong-positive pixels, min.=minutes, PPC=positive pixel count.

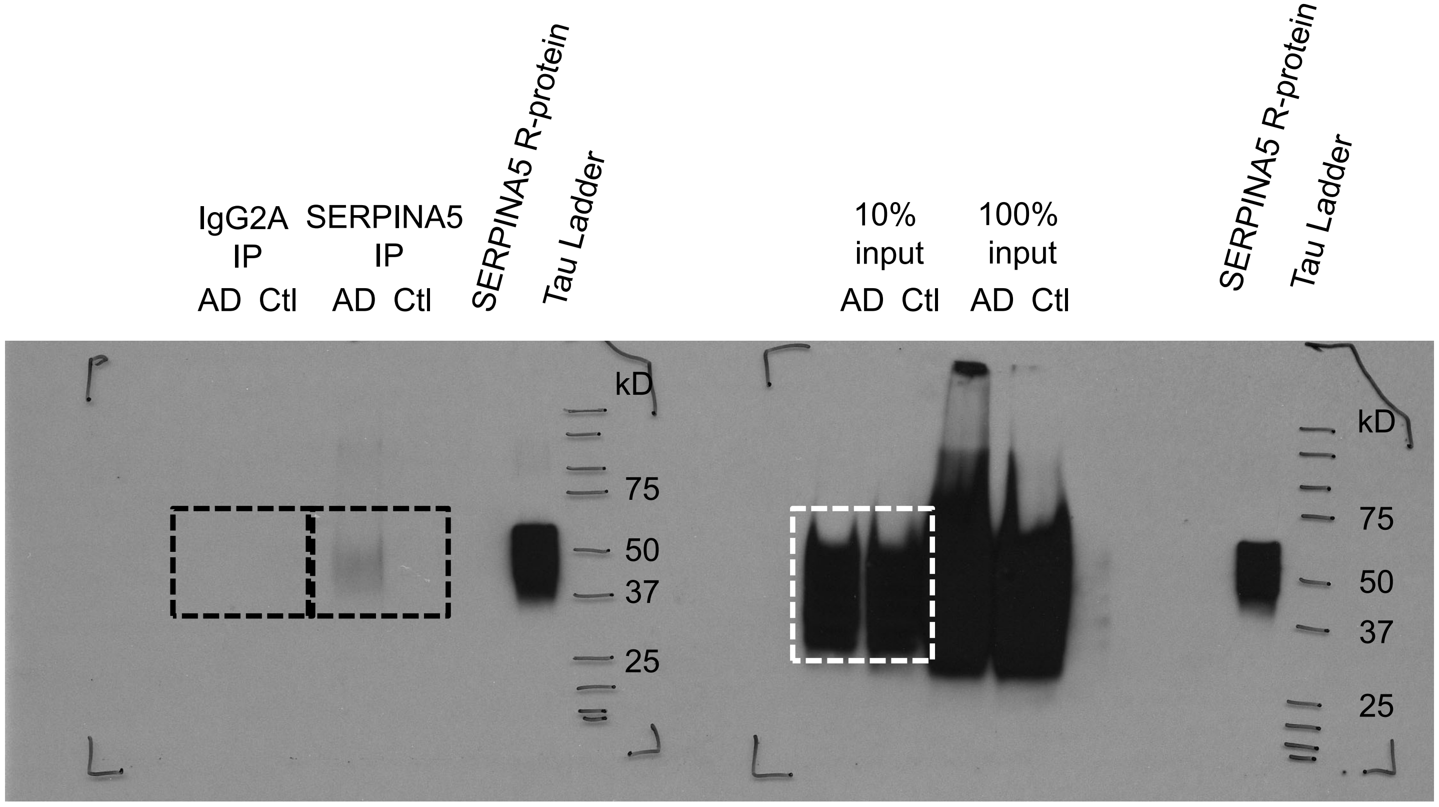

Supplementary Fig. 19 | Raw images of western blots from co-IP of hippocampus. Included are the original, uncropped images of the SERPINA5 immunoprecipitation and tau (E1 antibody) immunoblot shown from hippocampus in Fig. 6g after chemiluminescence exposure for 30 seconds. Note that the input represents the total homogenate before IP. Tissue was sampled from frozen hippocampi of a 73 year old male control (Braak=I, Thal=0) and an 86 year old male AD case (Braak=V, Thal=5). Acronyms: AD=Alzheimer's Disease, C=control, IP= immunoprecipitation.

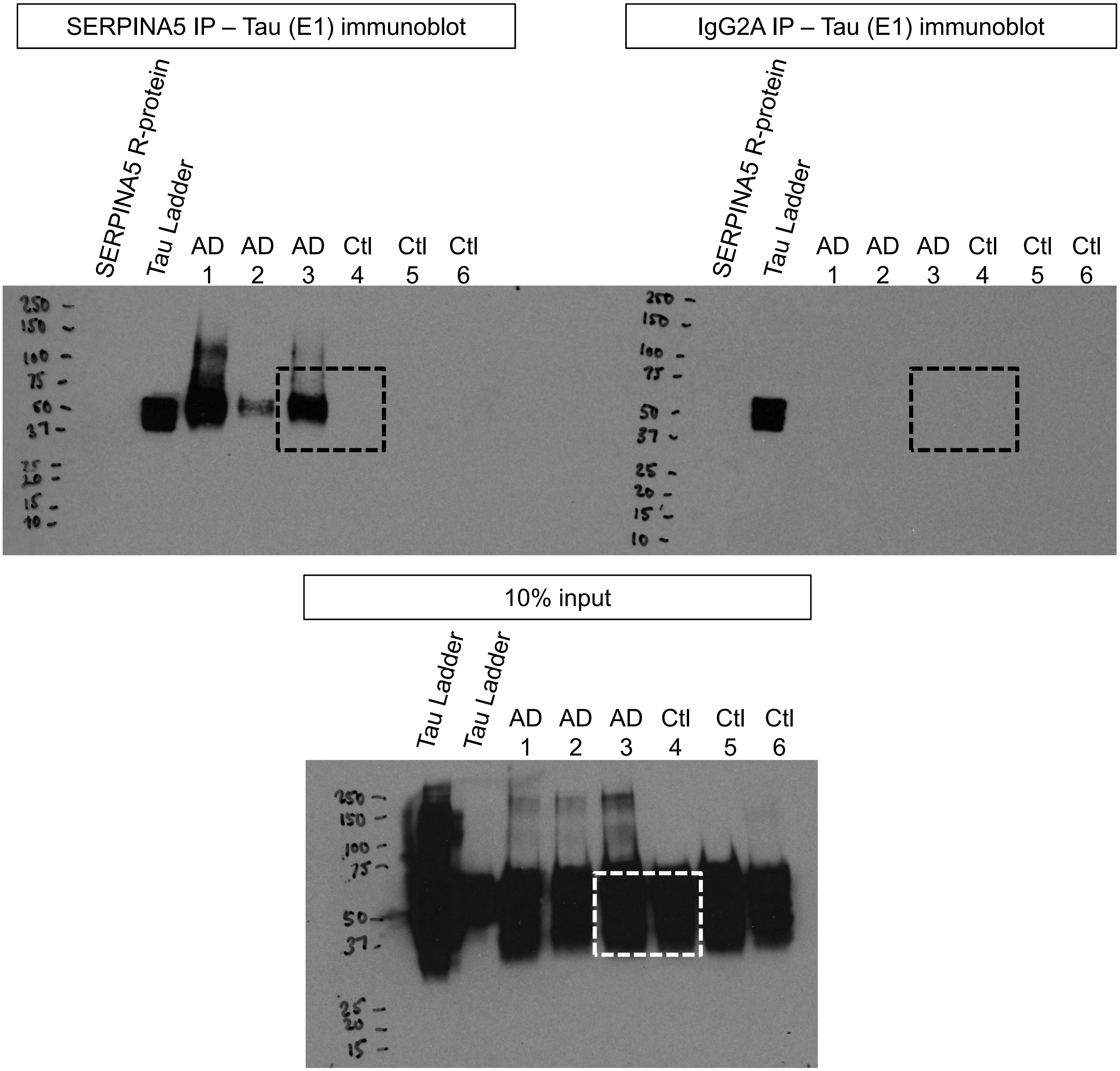

Supplementary Fig. 20 | Raw images of western blots from co-IP of frontal cortex. Included are the original, uncropped images of the SERPINA5 immunoprecipitation and tau (E1 antibody) immunoblot shown from frontal cortex in Fig. 6h after chemiluminescence exposure for 30 seconds. Note that the input represents the total homogenate before IP (exposure time 10 seconds). Tissue was sampled from frozen frontal cortices from three AD cases (#1 64 year old female [Braak=VI, Thal=5]; #2 60 year old female [Braak=VI, Thal=4]; #3 68 year old female [Braak VI; Thal 4]) and three controls (#4 75 year old female [Braak=I, Thal=0]; #5 78 year old male [Braak=II, Thal=1]; #6 96 year old female [Braak=II, Thal=3]). Acronyms: AD=Alzheimer's Disease, Ctl=control, IP= immunoprecipitation.

Supplementary Results 1 | Random Forest Importance Measures

Fig. 4a-b: Control versus all AD subtypes							
variable	mean_min_depth	no_of_nodes	accuracy_decrease	gini_decrease	no_of_trees	times_a_root	p_value
**SERPINA5**	**2.1797**	**309**	**0.0147**	**3.3182**	**256**	**41**	**<0.0001**
**RYBP**	**2.5852**	**246**	**0.0083**	**3.1178**	**209**	**44**	**<0.0001**
**SLC38A2**	**2.7758**	**236**	**0.0113**	**3.3811**	**196**	**54**	**<0.0001**
**FEM1B**	**3.2232**	**214**	**0.0038**	**1.9006**	**187**	**20**	**<0.0001**
**PYDC1**	**3.2656**	**262**	**0.0018**	**1.5229**	**206**	**6**	**<0.0001**
RELA	3.2908	209	0.0057	2.4203	182	46	0.0001
LCOR	3.3862	207	0.0051	2.2486	175	37	0.0001
DAXX	3.5147	185	0.0045	2.1183	160	36	0.0232
DNAI1	3.6677	211	0.0029	1.5603	174	3	<0.0001
OR7A5	3.6764	191	0.0078	1.4627	170	15	0.0072
DNAAF1	3.782	173	0.0033	1.4835	153	14	0.1436
CTCF	3.8684	173	0.0058	1.7211	151	24	0.1436
IFITM2	3.8903	211	0.0022	1.1026	174	1	<0.0001
MAPK15	4.0471	195	0.0027	1.1039	155	0	0.003
CAV1	4.1811	177	-0.0001	0.9606	150	0	0.0845
MAX	4.1876	157	0.0037	1.1818	139	11	0.5811
NCOA1	4.2112	167	0.0046	1.3378	138	10	0.2765
DST	4.2156	154	0.003	1.2361	136	16	0.6726
RP11.81K2.1	4.226	150	0.0002	1.0543	125	17	0.7803
FOXO4	4.2947	148	0.0006	0.9129	132	11	0.8256
TAC1	4.3413	151	0.0023	0.9947	133	7	0.7553
RBBP7	4.3748	155	0.0019	0.8123	140	1	0.6429
LRRC48	4.4197	155	0.0023	0.9321	132	3	0.6429
CXCL1	4.4378	153	0.0012	0.894	138	4	0.7014
ANGPT2	4.4806	135	0.0032	1.0137	122	14	0.9784
DYDC2	4.4813	150	0.002	0.961	136	0	0.7803
CNOT8	4.5519	135	0.0035	0.7853	118	7	0.9784
RBP1	4.5599	125	0.0003	0.6418	117	1	0.998
RAD52	4.5845	122	0.0011	0.7876	112	6	0.9992
ALOX15B	4.6165	130	0.0021	0.808	109	12	0.9928
ERBB2IP	4.6771	129	0.0018	0.77	117	2	0.9943
BCL2	4.7088	130	0.0021	0.8518	115	13	0.9928
PSEN2	4.7486	126	0.0003	0.6329	112	1	0.9974
ATR	4.7665	133	0.0006	0.637	119	0	0.9858
PPM1D	4.7688	117	-0.0001	0.7	109	4	0.9998
MAGED1	4.7872	126	0.001	0.6242	114	2	0.9974
SIPA1	4.7913	125	0.0021	0.6497	113	1	0.998
INSR	4.8254	111	0.0017	0.5754	101	3	1
EML4	4.8337	110	-0.0003	0.5623	99	1	1
CDKN2C	4.8459	113	0.0008	0.7107	97	3	1
SUN2	4.9796	102	0.0003	0.5637	93	4	1
SIRT1	4.9902	110	0.0009	0.5715	98	4	1
IRS2	5.0862	98	0.0002	0.5796	88	1	1
NOS1	5.1311	89	0	0.418	80	0	1

Supplementary Results 1 | Random Forest Importance Measures (continued)

Supplementary Data Fig. 8a: Representative Phenotype (Control versus Typical AD)							
variable	mean_min_depth	no_of_nodes	accuracy_decrease	gini_decrease	no_of_trees	times_a_root	p_value
**DNAAF1**	**2.1994**	**189**	**0.0093**	**2.2345**	**169**	**28**	**<0.0001**
**SERPINA5**	**2.3181**	**194**	**0.0078**	**1.8661**	**172**	**25**	**<0.0001**
**PYDC1**	**2.4054**	**221**	**0.0031**	**1.3465**	**185**	**2**	**<0.0001**
**RYBP**	**2.5101**	**178**	**0.008**	**2.1671**	**158**	**37**	**<0.0001**
**SLC38A2**	**2.5743**	**164**	**0.0121**	**2.4328**	**142**	**48**	**0.0018**
DNAI1	2.5764	188	0.0075	1.5829	162	9	<0.0001
NCOA1	2.5926	186	0.0096	1.7209	162	15	<0.0001
FEM1B	2.848	166	0.0073	1.6579	144	25	0.0011
LCOR	2.8938	154	0.0074	1.7766	133	33	0.0188
CTCF	2.9857	151	0.0096	1.8161	133	33	0.0339
DYDC2	3.0242	177	0.0057	1.3793	149	3	<0.0001
RELA	3.0288	152	0.0081	1.5441	136	28	0.028
MAPK15	3.0806	161	0.0022	1.3591	141	14	0.0039
DAXX	3.3025	124	0.0074	1.6102	113	31	0.7024
OR7A5	3.4147	146	0.0069	1.0304	131	12	0.0807
CAV1	3.5064	153	0.0004	0.8063	134	1	0.023
RP11.81K2.1	3.5332	127	0.0058	1.1257	112	22	0.6032
TAC1	3.6171	134	0.0024	0.945	123	8	0.3595
PSEN2	3.6283	135	0.0026	1.0294	117	13	0.3272
ALOX15B	3.8125	123	0.0021	0.8321	111	13	0.7329
MAGED1	3.843	124	0.0034	0.6378	113	4	0.7024
CXCL1	3.845	120	0.0005	0.7816	111	7	0.8144
SIPA1	3.9122	121	0.0024	0.6509	103	3	0.789
PPM1D	3.9152	123	0.0032	0.7126	111	7	0.7329
LRRC48	3.9464	118	0.0022	0.8817	104	9	0.8593
IFITM2	3.9751	120	0.0011	0.5677	105	0	0.8144
EML4	4.0078	114	0.0002	0.6264	102	4	0.9259
RBP1	4.0205	112	-0.0004	0.6672	100	4	0.9486
CNOT8	4.0242	105	0.0013	0.6609	99	7	0.989
ANGPT2	4.0928	107	0.0024	0.7651	98	9	0.9822
RBBP7	4.1972	107	0.0006	0.5529	99	1	0.9822
CDKN2C	4.1981	95	0.002	0.7694	87	9	0.9994
ERBB2IP	4.2352	103	0.0028	0.6157	96	6	0.9935
FOXO4	4.2506	88	0.0005	0.6509	83	7	1
RAD52	4.2573	96	-0.0005	0.592	90	3	0.9992
BCL2	4.2696	103	0.0013	0.524	94	2	0.9935
MAX	4.272	98	0.0019	0.6626	86	5	0.9985
INSR	4.2837	108	0.0015	0.5619	99	4	0.9776
DST	4.3302	88	0.0022	0.6232	79	5	1
ATR	4.3581	108	0.0011	0.4717	92	0	0.9776
IRS2	4.4921	85	0.0011	0.4139	82	0	1
SUN2	4.5425	89	0.0008	0.427	83	0	0.9999
SIRT1	4.5613	81	0.0006	0.5007	72	4	1
NOS1	4.771	66	-0.0008	0.363	62	0	1

Supplementary Results 1 | Random Forest Importance Measures (continued)

Supplementary Data Fig. 8a: Extreme Phenotype (Hippocampal sparing AD versus Limbic predominant

variable	mean_min_depth	no_of_nodes	accuracy_decrease	gini_decrease	no_of_trees	times_a_root	p_value
**FEM1B**	**1.5758**	**228**	**0.03**	**2.9679**	**198**	**59**	**<0.0001**
**ERBB2IP**	**2.9319**	**167**	**0.0123**	**1.3556**	**147**	**29**	**<0.0001**
**SERPINA5**	**2.9669**	**169**	**0.0045**	**1.404**	**152**	**23**	**<0.0001**
**LRRC48**	**3.0664**	**168**	**0.0064**	**1.3735**	**145**	**28**	**<0.0001**
**RP11.81K2.1**	**3.294**	**156**	**0.0047**	**1.2357**	**140**	**12**	**0.0004**
CAV1	3.4272	155	0.0054	1.0236	142	16	0.0006
MAPK15	3.4544	144	0.0047	1.2094	128	28	0.0109
RYBP	3.4791	137	0.0094	1.1538	122	26	0.0466
RAD52	3.4945	150	0.0046	1.1268	133	20	0.0024
ATR	3.5521	136	0.0005	1.057	119	28	0.056
RBP1	3.6641	134	0.0009	1.0091	120	24	0.0791
LCOR	3.774	120	0.0053	0.9222	108	23	0.4454
SIPA1	3.8198	113	0.0022	0.9982	103	32	0.6978
CNOT8	3.8233	128	0.0017	0.818	112	15	0.1919
BCL2	3.846	114	0.003	0.9601	104	22	0.6641
DST	3.9445	123	0.0003	0.7234	112	8	0.3394
IRS2	4.0088	131	-0.0024	0.7275	116	2	0.1267
OR7A5	4.0451	126	-0.0011	0.7052	117	2	0.2454
IFITM2	4.0865	134	0.0028	0.769	118	5	0.0791
DAXX	4.0894	111	0.0017	0.6542	105	0	0.7604
ALOX15B	4.0957	111	0.0007	0.6706	101	8	0.7604
SLC38A2	4.2233	108	-0.0014	0.6634	97	7	0.8399
CDKN2C	4.2473	115	0.0019	0.6659	101	7	0.6291
CTCF	4.2684	113	0.0004	0.6696	102	0	0.6978
PYDC1	4.292	122	0.0013	0.6095	111	6	0.3737
DNAAF1	4.3002	115	-0.0007	0.5362	109	2	0.6291
PPM1D	4.3432	108	0.001	0.5471	101	4	0.8399
CXCL1	4.361	98	-0.001	0.5225	93	4	0.9755
NOS1	4.3761	105	-0.0006	0.5852	93	6	0.9003
EML4	4.3862	96	0.001	0.4927	93	3	0.9849
RELA	4.4074	104	0.0013	0.5437	94	0	0.9162
FOXO4	4.4163	91	-0.0013	0.5003	82	7	0.9962
NCOA1	4.4491	101	-0.0021	0.5644	90	3	0.9528
TAC1	4.4825	93	-0.0025	0.5216	88	3	0.9932
INSR	4.5312	96	-0.0007	0.5633	86	5	0.9849
SUN2	4.5375	98	-0.0006	0.5555	82	7	0.9755
MAGED1	4.5571	87	0.0005	0.4735	76	3	0.999
MAX	4.5615	88	0.0009	0.4183	86	1	0.9985
ANGPT2	4.5757	94	0	0.4731	85	2	0.991
RBBP7	4.6168	82	0.0011	0.4963	75	4	0.9998
SIRT1	4.6256	87	-0.0004	0.4486	79	3	0.999
PSEN2	4.6417	89	-0.0003	0.5317	80	8	0.998
DYDC2	4.7601	81	-0.0009	0.3979	77	0	0.9999
DNAI1	4.8302	74	-0.0002	0.3635	71	5	1

Supplementary Results 1 | Random Forest Importance Measures (continued)
Column descriptions

**Column**	**Description**
accuracy_decrease (classification)	mean decrease of prediction accuracy after X j Xj is permuted
gini_decrease (classification)	mean decrease in the Gini index of node impurity (i.e. increase of node purity) by splits on X j Xj
mse_increase (regression)	mean increase of mean squared error after X j Xj is permuted
node_purity_increase (regression)	mean node purity increase by splits on X j Xj , as measured by the decrease in sum of squares
e. mean_minimal_depth	mean minimal depth calculated in one of three ways specified by the parameter mean_sample
no_of_trees	total number of trees in which a split on X j Xj occurs
no_of_nodes	total number of nodes that use X j Xj for splitting (it is usually equal to no_of_trees if trees are shallow)
times_a_root	total number of trees in which X j Xj is used for splitting the root node (i.e., the whole sample is divided into two based on the value of X j Xj)
p_value	p-value for the one-sided binomial test using the following distribution

For description of other metrics included in these tables, please refer to:
https://cran.r-project.org/web/packages/randomForestExplainer/vignettes/randomForestExplainer.html

Supplementary 1: Random Forest analyses were applied to examine the “importance” of gene expression changes when discriminating between groups. The main analysis examined differences between controls versus all AD subtypes with sub-analyses of the representative phenotype and extreme phenotype. Judicious examination of the mean minimum depth and times a root was ranked to facilitate identifying the top 5 genes.

Supplementary Results 2 | AMP-AD Top 5 Gene Comparison

Mayo-TCX: Mayo Clinic Temporal Cortex (syn3163039, TCX)

GeneName	log2FC	FDR	p-value	meanCQN.control	meanCQN.AD	AvgMappedReads	Direction
SERPINA5	2.1	0.000040	0.00000085	-0.15	1.5	103	Upregulated
RYBP	0.0049	1.0	0.95	5.6	5.7	3165	Upregulated
SLC38A2	1.0	0.0000015	0.0000000086	6.9	7.7	9841	Upregulated
FEM1B	-0.10	0.24	0.072	6.3	6.3	5092	Downregulated
PYDC1	-0.78	0.00077	0.000039	3.2	3.2	77	Downregulated

MSBB-BM22: Mt. Sinai Brain Bank Superior Temporal Cortex (syn20801188, BM22)

GeneName	log2FC	FDR	p-value	meanCQN.control	meanCQN.AD	AvgMappedReads	Direction
SERPINA5	1.0	0.095	0.00040	-0.57	0.23	21	Upregulated
RYBP	0.14	0.34	0.029	4.3	4.4	1024	Upregulated
SLC38A2	0.35	0.27	0.015	6.3	6.6	4105	Upregulated
FEM1B	-0.038	0.82	0.40	6.0	6.0	3173	Downregulated
PYDC1	-0.27	0.59	0.15	1.2	0.89	17	Downregulated

MSBB-BM36: Mt. Sinai Brain Bank Superior Temporal Cortex (syn20801188, BM36)

GeneName	log2FC	FDR	p-value	meanCQN.control	meanCQN.AD	AvgMappedReads	Direction
SERPINA5	2.0	0.0016	0.0000030	-1.1	0.81	31	Upregulated
RYBP	0.17	0.051	0.0015	4.5	4.6	1077	Upregulated
SLC38A2	0.29	0.28	0.039	6.3	6.6	3710	Upregulated
FEM1B	-0.10	0.21	0.022	6.2	6.0	3180	Downregulated
PYDC1	-0.46	0.066	0.0024	2.2	1.7	22	Downregulated

Crist et al. (Mayo Clinic Hippocampus)

GeneName	log2FC	FDR	p-value	Mean.Ctrl	Mean.Typical	AvgMappedReads	Direction
SERPINA5	1.7	0.0061	0.0000041	-0.87	0.68	220	Upregulated
RYBP	0.28	0.12	0.0027	3.2	3.4	3659	Upregulated
SLC38A2	0.93	0.0050	0.0000026	4.7	5.5	13845	Upregulated
FEM1B	0.20	0.17	0.0062	4.5	4.7	9333	Upregulated
PYDC1	-1.0	0.21	0.0089	0.36	-0.87	18	Downregulated

Supplementary Results 2: To further examine the relevance of the top 5 genes outside the hippocampus, we investigated three AMP-AD validation datasets. We present differential expression analysis of *SERPINA5, RYBP, SLC38A2, FEM1B, and PYDC1* in the temporal cortex (Mayo-TCX syn3163039[25,38], MSBB-BM22 syn20801188[39]) and the parahippocampal gyrus (MSBB-BM36 syn20801188[39]), as well as a comparison to the current study. *SERPINA5* and *SLC38A2* were significantly upregulated across all three validation datasets. *RYBP* was significantly upregulated in two of the three validation datasets. *FEM1B* performed in the opposite direction compared to the current study. *PYDC1* was significantly downregulated across all three validation datasets.

Supplementary Results 3 | Neuner Review[78] Literature Genes

GeneName	GeneID	Step 4: Group-wise difference controls and AD subtypes p-value must be <0.05	Step 4: Mono-tonically directed	Step 4: Must be protein coding	Step 4: Neuropathologic measures fdr<0.25, p<0.05 Robustly associate in both analyses	Pass/Fail summary
ABCA7	ENSG00000064687	0.224432	No	Pass	no association	Fail, group-wise differences p>0.05
ABI3	ENSG00000108798	0.267804	No	Pass	no association	Fail, group-wise differences p>0.05
AC099552.4	ENSG00000217825	0.484519	Yes	Fail in Step 0	no association	Fail in Step 0 (low expressing)
ACE	ENSG00000159640	0.11499	Yes	Fail in Step 0	no association	Fail in Step 0 (low expressing)
ACE	ENSG00000264813	0.462424	No	Fail in Step 0	no association	Fail in Step 0 (low expressing)
ACSM1	ENSG00000166743	0.345316	No	Fail in Step 0	no association	Fail in Step 0 (low expressing)
ADAM10	ENSG00000137845	0.00676065	Yes	Pass	Ab39	Passed Step 4 criteria
ADAMTS1	ENSG00000154734	0.0281646	No	Pass	Ab39	Fail, not monotonically-directed
ADAMTS4	ENSG00000158859	0.302511	No	Pass	no association	Fail, group-wise differences p>0.05
AKAP9	ENSG00000127914	0.322729	No	Pass	no association	Fail, group-wise differences p>0.05
ALPK2	ENSG00000198796	0.199508	No	Fail in Step 0	no association	Fail in Step 0 (low expressing)
ANKMY2	ENSG00000106524	0.00086514	Yes	Pass	Braak; Thal; Ab39	Passed Step 4 criteria
ANKS4B	ENSG00000175311	0.0245938	Yes	Fail in Step 0	no association	Fail in Step 0 (low expressing)
APH1B	ENSG00000138613	0.315639	No	Pass	no association	Fail, group-wise differences p>0.05
APOE	ENSG00000130203	0.165588	No	Pass	no association	Fail, group-wise differences p>0.05
APP	ENSG00000142192	0.510598	No	Pass	no association	Fail, group-wise differences p>0.05
ATP5F1	ENSG00000116459	0.00322088	Yes	Pass	Braak; Thal; Ab39	Passed Step 4 criteria
B3GALT2	ENSG00000162630	0.345955	Yes	Pass	no association	Fail, group-wise differences p>0.05
BCKDK	ENSG00000103507	0.620264	Yes	Pass	no association	Fail, group-wise differences p>0.05
BDNF	ENSG00000176697	0.234211	No	Pass	no association	Fail, group-wise differences p>0.05
BIN1	ENSG00000136717	0.320193	No	Pass	no association	Fail, group-wise differences p>0.05
BLOC1S3	ENSG00000189114	0.254192	No	Pass	no association	Fail, group-wise differences p>0.05
BZRAP1-AS1	ENSG00000265148	0.415163	No	Fail	no association	Fail, group-wise differences p>0.05
BZW2	ENSG00000136261	0.154298	Yes	Pass	no association	Fail, group-wise differences p>0.05
C16orf62	ENSG00000103544	0.51216	No	Pass	no association	Fail, group-wise differences p>0.05
CASS4	ENSG00000087589	0.0271143	No	Pass	no association	Fail, not monotonically-directed
CD2AP	ENSG00000198087	0.817111	Yes	Pass	no association	Fail, group-wise differences p>0.05
CD33	ENSG00000105383	0.271216	No	Pass	no association	Fail, group-wise differences p>0.05
CELF1	ENSG00000149187	0.795317	No	Pass	no association	Fail, group-wise differences p>0.05
CLNK	ENSG00000109684	0.310674	No	Fail in Step 0	no association	Fail in Step 0 (low expressing)
CLU	ENSG00000120885	0.215988	Yes	Pass	no association	Fail, group-wise differences p>0.05
CNTN5	ENSG00000149972	0.120932	No	Pass	no association	Fail, group-wise differences p>0.05
CNTNAP2	ENSG00000174469	0.19226	Yes	Pass	no association	Fail, group-wise differences p>0.05
COBL	ENSG00000106078	0.770243	No	Pass	no association	Fail, group-wise differences p>0.05
CR1	ENSG00000203710	0.397434	No	Fail in Step 0	no association	Fail in Step 0 (low expressing)
CTDP1	ENSG00000060069	0.102563	No	Pass	no association	Fail, group-wise differences p>0.05
DAPK1	ENSG00000196730	0.140857	No	Pass	no association	Fail, group-wise differences p>0.05
DSG2	ENSG00000046604	0.249527	Yes	Fail in Step 0	no association	Fail in Step 0 (low expressing)
ECHDC3	ENSG00000134463	0.393534	No	Pass	no association	Fail, group-wise differences p>0.05
EPHA1	ENSG00000146904	0.131115	Yes	Fail in Step 0	no association	Fail in Step 0 (low expressing)
EXOC3L2	ENSG00000130201	0.617172	No	Fail in Step 0	no association	Fail in Step 0 (low expressing)
FAM63B	ENSG00000128923	0.292611	No	Pass	no association	Fail, group-wise differences p>0.05
FBP1	ENSG00000165140	0.80813	No	Pass	no association	Fail, group-wise differences p>0.05
FERMT2	ENSG00000073712	0.644659	No	Pass	no association	Fail, group-wise differences p>0.05
FRA10AC1	ENSG00000148690	0.269099	No	Pass	no association	Fail, group-wise differences p>0.05

Supplementary Results 3 | Neuner Review[78] Literature Genes continued

GeneName	GeneID	Step 4: Group-wise difference controls and AD subtypes p-value must be <0.05	Step 4: Mono-tonically directed	Step 4: Must be protein coding	Step 4: Neuropathologic measures fdr<0.25, p<0.05 Robustly associate in both analyses	Pass/Fail summary
GALNT7	ENSG00000109586	0.888093	No	Pass	no association	Fail, group-wise differences p>0.05
GDE1	ENSG00000006007	0.302725	No	Pass	no association	Fail, group-wise differences p>0.05
GLIS1	ENSG00000174332	0.143503	Yes	Pass	no association	Fail, group-wise differences p>0.05
GLIS3	ENSG00000107249	0.237899	No	Pass	no association	Fail, group-wise differences p>0.05
GMNC	ENSG00000205835	0.297753	Yes	Pass	no association	Fail, group-wise differences p>0.05
GPRC5B	ENSG00000167191	0.0518086	Yes	Pass	Ab39	Fail, group-wise differences p>0.05
GRIN2B	ENSG00000273079	0.398606	No	Pass	no association	Fail, group-wise differences p>0.05
HBEGF	ENSG00000113070	0.982263	No	Pass	no association	Fail, group-wise differences p>0.05
HESX1	ENSG00000163666	0.57992	No	Pass	no association	Fail, group-wise differences p>0.05
HLA-DRB1	ENSG00000196126	0.114551	No	Pass	no association	Fail, group-wise differences p>0.05
HLA-DRB5	ENSG00000198502	0.863747	No	Pass	no association	Fail, group-wise differences p>0.05
HS3ST1	ENSG00000002587	0.0416514	No	Pass	no association	Fail, not monotonically-directed
IL34	ENSG00000157368	0.419555	No	Pass	no association	Fail, group-wise differences p>0.05
INPP5D	ENSG00000168918	0.667486	No	Pass	no association	Fail, group-wise differences p>0.05
IQCK	ENSG00000174628	0.596658	Yes	Pass	no association	Fail, group-wise differences p>0.05
ITGA8	ENSG00000077943	0.00867443	No	Pass	Thal	Fail, not monotonically-directed
KAT8	ENSG00000103510	0.0231268	Yes	Pass	no association	Fail, no association with neuropath
KCNH6	ENSG00000173826	0.395575	No	Fail in Step 0	no association	Fail in Step 0 (low expressing)
KNOP1	ENSG00000103550	0.125756	No	Pass	no association	Fail, group-wise differences p>0.05
LRP6	ENSG00000070018	0.659092	No	Pass	no association	Fail, group-wise differences p>0.05
MARK4	ENSG00000007047	0.992413	No	Pass	no association	Fail, group-wise differences p>0.05
MEF2C	ENSG00000081189	0.158461	No	Pass	no association	Fail, group-wise differences p>0.05
MS4A2	ENSG00000149534	0.868523	No	Fail in Step 0	no association	Fail in Step 0 (low expressing)
MS4A4A	ENSG00000110079	0.802155	No	Pass	no association	Fail, group-wise differences p>0.05
MS4A6A	ENSG00000110077	0.097507	No	Pass	no association	Fail, group-wise differences p>0.05
MS4A6E	ENSG00000166926	0.0317935	No	Fail in Step 0	no association	Fail in Step 0 (low expressing)
NFATC1	ENSG00000131196	0.919537	Yes	Pass	no association	Fail, group-wise differences p>0.05
NME8	ENSG00000086288	0.23112	No	Fail in Step 0	no association	Fail in Step 0 (low expressing)
NOTCH3	ENSG00000074181	0.755962	No	Pass	no association	Fail, group-wise differences p>0.05
NYAP1	ENSG00000166924	0.963773	No	Pass	no association	Fail, group-wise differences p>0.05
OARD1	ENSG00000124596	0.286194	No	Pass	no association	Fail, group-wise differences p>0.05
OSTN	ENSG00000188729	0.168762	No	Fail in Step 0	no association	Fail in Step 0 (low expressing)
PCDH8	ENSG00000136099	0.549915	No	Pass	no association	Fail, group-wise differences p>0.05
PFDN1	ENSG00000113068	0.0902279	No	Pass	no association	Fail, group-wise differences p>0.05
PICALM	ENSG00000073921	0.254338	No	Pass	no association	Fail, group-wise differences p>0.05
PILRA	ENSG00000085514	0.923296	No	Pass	no association	Fail, group-wise differences p>0.05
PIP	ENSG00000159763	0.559166	No	Fail in Step 0	no association	Fail in Step 0 (low expressing)
PLCG2	ENSG00000197943	0.370988	No	Pass	no association	Fail, group-wise differences p>0.05
PLD3	ENSG00000105223	0.599286	No	Pass	no association	Fail, group-wise differences p>0.05
PRNP	ENSG00000171867	0.666178	No	Pass	no association	Fail, group-wise differences p>0.05
PSEN1	ENSG00000080815	0.79361	No	Pass	no association	Fail, group-wise differences p>0.05
PSEN2	ENSG00000143801	0.0154051	Yes	Pass	Thal; Ab39	Passed Step 4 criteria
PSMB8	ENSG00000204264	0.200604	No	Pass	no association	Fail, group-wise differences p>0.05
PTK2B	ENSG00000120899	0.487029	No	Pass	no association	Fail, group-wise differences p>0.05
PVRL2	ENSG00000130202	0.77427	Yes	Pass	no association	Fail, group-wise differences p>0.05

Supplementary Results 3 | Neuner Review[78] Literature Genes continued

GeneName	GeneID	Step 4: Group-wise difference controls and AD subtypes p-value must be <0.05	Step 4: Mono-tonically directed	Step 4: Must be protein coding	Step 4: Neuropathologic measures fdr<0.25, p<0.05 Robustly associate in both analyses	Pass/Fail summary
RBFOX1	ENSG00000078328	0.526243	No	Pass	no association	Fail, group-wise differences p>0.05
RIN3	ENSG00000100599	0.0346903	No	Pass	no association	Fail, not monotonically-directed
SCIMP	ENSG00000161929	0.775518	Yes	Fail in Step 0	no association	Fail in Step 0 (low expressing)
SERPINB1	ENSG00000021355	0.858301	No	Pass	no association	Fail, group-wise differences p>0.05
SLC10A2	ENSG00000125255	0.636976	No	Fail in Step 0	no association	Fail in Step 0 (low expressing)
SLC24A4	ENSG00000140090	0.252707	Yes	Pass	no association	Fail, group-wise differences p>0.05
SLTM	ENSG00000137776	0.280178	No	Pass	no association	Fail, group-wise differences p>0.05
SORL1	ENSG00000137642	0.0109846	No	Pass	no association	Fail, not monotonically-directed
SPI1	ENSG00000066336	0.145055	No	Pass	no association	Fail, group-wise differences p>0.05
SPPL2A	ENSG00000138600	0.467655	No	Pass	no association	Fail, group-wise differences p>0.05
STYX	ENSG00000198252	0.22876	No	Pass	no association	Fail, group-wise differences p>0.05
TAP2	ENSG00000204267	0.0177813	No	Pass	no association	Fail, not monotonically-directed
TF	ENSG00000091513	0.848483	No	Pass	no association	Fail, group-wise differences p>0.05
TOMM40	ENSG00000130204	0.11573	No	Pass	no association	Fail, group-wise differences p>0.05
TREM2	ENSG00000095970	0.620013	No	Pass	no association	Fail, group-wise differences p>0.05
UNC5C	ENSG00000182168	0.628214	No	Pass	no association	Fail, group-wise differences p>0.05
USP6NL	ENSG00000148429	0.0676954	No	Pass	no association	Fail, group-wise differences p>0.05
WWOX	ENSG00000186153	0.632314	No	Pass	no association	Fail, group-wise differences p>0.05
ZNF232	ENSG00000167840	0.151841	No	Pass	no association	Fail, group-wise differences p>0.05
SUZ12P1	--	--	--	--	--	Not identified in our dataset
PPB	--	--	--	--	--	Not identified in our dataset
NCR2	--	--	--	--	--	Not identified in our dataset
BZRAP-AS1	--	--	--	--	--	Not identified in our dataset

Supplementary Results 3: A contemporary literature-based gene set derived from Neuner, et al.[78] was additionally examined using the translational neuropathology approach from Step 4. The goal was to provide an updated gene set and put into context of the current study. Three additional genes met inclusion criteria, which will be included in future studies: *ADAM10, ANKMY2, and ATP5F1.*
