# Supplementary material for: Transcriptomic analysis to identify genes associated with selective hippocampal vulnerability in Alzheimer’s disease
Source: Nat Commun. 2021 Apr 19;12:2311. doi: 10.1038/s41467-021-22399-3 (PMC8055900; doi:10.1038/s41467-021-22399-3)
Supplement: Supplementary file 3 — Reporting Summary [file 41467_2021_22399_MOESM3_ESM.pdf]

nature research

Corresponding author(s): Melissa E. Murray, PhD

Last updated by author(s): 1/30/2021

Reporting Summary

Nature Research wishes to improve the reproducibility of the work that we publish. This form provides structure for consistency and transparency in reporting. For further information on Nature Research policies, see Authors & Referees and the Editorial Policy Checklist .

Statistics

For all statistical analyses, confirm that the following items are present in the figure legend, table legend, main text, or Methods section.

n/a	Confirmed	
☐	☒	The exact sample size (*n*) for each experimental group/condition, given as a discrete number and unit of measurement
☐	☒	A statement on whether measurements were taken from distinct samples or whether the same sample was measured repeatedly
☐	☒	The statistical test(s) used AND whether they are one- or two-sided<br>*Only common tests should be described solely by name; describe more complex techniques in the Methods section.*
☐	☒	A description of all covariates tested
☐	☒	A description of any assumptions or corrections, such as tests of normality and adjustment for multiple comparisons
☐	☒	A full description of the statistical parameters including central tendency (e.g. means) or other basic estimates (e.g. regression coefficient) AND variation (e.g. standard deviation) or associated estimates of uncertainty (e.g. confidence intervals)
☐	☒	For null hypothesis testing, the test statistic (e.g. *F*, *t*, *r*) with confidence intervals, effect sizes, degrees of freedom and *P* value noted<br>*Give P values as exact values whenever suitable.*
☒	☐	For Bayesian analysis, information on the choice of priors and Markov chain Monte Carlo settings
☐	☒	For hierarchical and complex designs, identification of the appropriate level for tests and full reporting of outcomes
☒	☐	Estimates of effect sizes (e.g. Cohen's *d*, Pearson's *r*), indicating how they were calculated

Our web collection on statistics for biologists contains articles on many of the points above.

Software and code

Policy information about availability of computer code

Data collection	RNA prep: NanoDrop 1000, RNA 6000 Nano Chips on the 2100 bioanalyzer<br>RNA-seq: HiSeq2500, MAPRSeq v2.0<br>Digital pathology: Aperio AT2 scanner, Aperio ImageScope v12.4.2.7000 and v12.4.3.7001<br>Immunofluorescence: LSM 880 confocal microscope<br>Western blot: Kodak X-OMAT 2000 Processor
Data analysis	RNA-seq: MAPRSeq v2.0, TopHat v2.0.12, Subread tool kit v1.4.4, cqn package v1.16.0, Partek® Genomics Suite® software v6.6, edgeR package v3.12.0, GeneGo MetaCore Build 6.24.67895<br>NanoString: NanoString nCounter v1.0.84, R package OSAT v1.18, NanoStringNorm package v1.2.1<br>Machine Learning: randomForest package v4.6-12, randomForestExplainer v0.9

For manuscripts utilizing custom algorithms or software that are central to the research but not yet described in published literature, software must be made available to editors/reviewers. We strongly encourage code deposition in a community repository (e.g. GitHub). See the Nature Research guidelines for submitting code & software for further information.

Data

Policy information about availability of data

All manuscripts must include a data availability statement. This statement should provide the following information, where applicable:
- Accession codes, unique identifiers, or web links for publicly available datasets
- A list of figures that have associated raw data
- A description of any restrictions on data availability

All requests for raw and analyzed data and related materials, excluding programming code, will be reviewed by Mayo Clinic's Legal Department and Mayo Clinic Ventures to verify whether each request is subject to any intellectual property or confidentiality obligations. Requests for patient-related data not included in the paper will not be considered. Any data and materials that can be shared will be released via a Data Use/Share Agreement or Material Transfer Agreement. The

Accelerating Medicines Partnership (AMP-AD) data in this manuscript are available via the AD Knowledge Portal (https://adknowledgeportal.synapse.org). Ensemble database queries used to examine gene names from RNA-Seq files relied upon http://dec2015.archive.ensembl.org/index.html and for contemporary literature-based gene set derived from Neuner, et al.78 http://aug2020.archive.ensembl.org/index.html was used. The results published here are in whole or in part based on data obtained from the AD Knowledge Portal (https://adknowledgeportal.synapse.org/, Synapse IDs: syn17010685, syn3163039, syn20801188).

Field-specific reporting

Please select the one below that is the best fit for your research. If you are not sure, read the appropriate sections before making your selection.

☒ Life sciences ☐ Behavioural & social sciences ☐ Ecological, evolutionary & environmental sciences

For a reference copy of the document with all sections, see nature.com/documents/nr-reporting-summary-flat.pdf

Life sciences study design

All studies must disclose on these points even when the disclosure is negative.

Sample size

No explicit calculations were performed to determine sample size. Our goal was to analyze brain tissue firstly using RNA-sequencing (RNA-Seq) to prioritize genes for validation using NanoString in a more-than-doubled cohort. To focus our investigation, we limited our analyses to AD cases lacking significant co-existing pathologies. RNA-seq required a high RIN (>7.0), thus we selected brains with highest quality tissue with equal number in extreme phenotypes (hippocampal sparing [n=10] and limbic predominant [n=10]) and a larger typical AD cohort as these are more representative of AD. To ensure ability to double controls analyzed in NanoString, half of the controls n=15) were analyzed with RNA-sequencing. To confirm NanoString gene expression measures associated with gene expression derived from RNA-seq, the RNA-seq cohort was included in NanoString analyses. Cases with DV300 >50% were included in the more-than-doubled NanoString cohort. These numbers of samples was sufficient to perform a confident data analysis.

Data exclusions

All data exclusions are explicitly stated and explained in the Results and Materials and Methods section of the paper. See summary below.

RNA-quality exclusions: Following RNA extraction, three brains were excluded for poor quality RNA. Of the remaining 32 controls, 15 with a RIN ≥7.0 were selected for RNA-Seq analyses.

Case Selection exclusions: In order to perform gene expression studies, we excluded 534 brains that did not have available frozen tissue for dissection. In an effort to reduce neurobiologic heterogeneity in the remaining 1,339 AD cases, we further excluded cases with co-existing pathologies (e.g. Lewy body pathology, tauopathies, tumors). Of the remaining 582 AD cases, we additionally excluded for significant cerebrovascular disease (e.g. infarcts, hippocampal ischemia). Of the remaining 402 AD cases, there were 16 AD cases that self-reported as non-Hispanic white, including 2 African-American/black decedents and 14 Hispanic/Latino decedents. One individual had an unknown ethnoracial status. These 17 AD cases were excluded as each of the AD subtypes was not represented and control brains were not available for matching.

NanoString analysis exclusions: Upon review of log2 transformation of normalized NanoString gene expression, we identified seven AD cases that would require a 2.5 fold adjustment with respect to housekeeping gene correction (Extended Data Fig. 5). An additional case was excluded due to low gene expression counts overall. These eight cases were excluded from further analyses, resulting in a final NanoString cohort of 182 AD cases and controls (Fig. 1b). These exclusion criteria were not pre-established.

Replication

Top 44 genes from RNA-Seq were replicated using NanoString in a larger cohort. Although not shown, the immunoprecipitation experiment was performed in an additional 2 AD samples in frontal cortex and yielded similar results. Likewise, immunofluorescent staining of SERPINA5 and tau was performed in triplicate and yielded similar results.

Randomization

For NanoString nCounter analysis, R package OSAT63 v1.18 was used to perform blocking randomization on 192 samples across all four groups (control, hippocampal sparing AD, typical AD and limbic predominant AD), sex (male or female), age (55-103) and RIN (4.6-8.9). Each NanoString plate had 2 X 6 wells and held 12 samples. Thus, age and RIN were stratified into 15 levels each. Samples were assigned to 15 plates in such a way that 13 plates held 12 samples each and 2 plates held 11 samples each. Samples were distributed in each plate as evenly as possible according to an objective function.

Blinding

RNA was extracted blinded to the AD subtype and demographic data. Digital pathology tracing and analysis was performed blinded to the AD subtype and demographic data. SERPINA5-positive neurofibrillary tangle counts was performed blinded to the AD subtype and demographic data. Although no blinding was performed on immunofluorescent staining or immunoprecipitation experiments, great care was taken to treat all samples in a consistent manner using the same master mixes and incubation times for all slides/samples. The size of the hippocampus is visible during immunofluorescent staining and extent of pathology readily apparent during evaluation on confocal, which reveals disease status. To facilitate interpretation of triplicates for co-immunoprecipitation experiments, AD cases and controls were loaded alongside one another.

Reporting for specific materials, systems and methods

We require information from authors about some types of materials, experimental systems and methods used in many studies. Here, indicate whether each material, system or method listed is relevant to your study. If you are not sure if a list item applies to your research, read the appropriate section before selecting a response.

Materials & experimental systems

n/a	Involved in the study
☐	☒ Antibodies
☒	☐ Eukaryotic cell lines
☒	☐ Palaeontology
☒	☐ Animals and other organisms
☐	☒ Human research participants
☒	☐ Clinical data

Methods

n/a	Involved in the study
☒	☐ ChIP-seq
☒	☐ Flow cytometry
☒	☐ MRI-based neuroimaging

Antibodies

Antibodies used

Immunohistochemical studies
Antibody, Supplier, Catalog #, Dilution, Antigen Retrieval are all shown below:
CP13 Peter Davies gift n/a 1:1000 30 min. steam in dH20
Ab39 Shu-Hui Yen gift n/a 1:350 30 min. steam in dH20
33.1.1 Pritam Das gift n/a 1:1000 30 min. in 98% Formic acid followed by 30 min. steam in dH20
GFAP Biogenex MU020-UC 1:5000 30 min. steam in dH20
CD34 Abcam Ab81289 1:25 30 min. steam in dH20
CD68 Dako M0814 1:1000 30 min. steam in dH20
SERPINA5 R&D MAB1266 1:100 30 min. steam in dH20

Immunofluorescence studies
Antibody, manufacturer, catalog number, dilution factor, antigen retrieval buffer, and lot number with lot specific concentrations are all shown below:
GFAP ABCAM ab33922 1:800 H2O Lot # GR3240236-5 (0.55 mg/mL)
MAP2 Abcam ab32454 1:200 H2O Lot # GR3199625-1 (1.0 mg/mL)
IBA1 Abcam ab178847 1:100 Citrate Lot # GR3229566-4 (0.63 mg/mL)
CD68 Cell Signaling 76437 1:400 Citrate Lot # 1 (0.292 mg/mL)
CD34 Abcam ab81289 1:200 H2O Lot # GR3240236-5 (0.55 mg/mL)
OLIG2 Abcam ab109186 1:100 Citrate Lot # GR3251505-5 (0.12 mg/mL)
SERPINA5 R&D MAB1266 1:100 H2O Lot # HQR0218101 or JCG0319031 (1 ug/uL)
Tau E1 Petrucelli Laboratory1 1:1000 H2O Lot # unknown
Tau pS396 Abcam ab109390 1:1000 Lot # GR3254929-4 (0.104 mg/mL)
Tau E178 Abcam ab32057 1:1000 Lot # GR303639-22 (0.091 mg/mL)
IgG2A R&D MAB003 1:100 Lot # MV0918031 (1 ug/uL)

Secondary Antibodies
AlexaFluor488 Goat α Mouse Invitrogen A11001 1:500 Lot # 1939600
AlexaFluor568 Goat α Rabbit Invitrogen A11011 1:500 Lot # 2013083

Validation

SERPINA5 (R&D MAB1266) was tested by the manufacturer for western blot, immunoprecipitation, and direct ELISA using recombinant human SerpinA5 (R&D 1266-PI). M. Murray lab verified that SERPINA5 (R&D MAB1266) peptide sequence (immunogen) does not cross-react with MAPT sequence.
Additional experiments performed to validate antibody included:
1) immunohistochemistry and western blot using human liver (where SERPINA5 is highly expressed) and human hippocampus
2) western blot using recombinant human SERPINA5 (R&D 1266-PI-010, Lot# HQR0218101)
3) immunohistochemistry and immunofluorescence staining (in human hippocampus) probing with isotype control only (IgG2A R&D MAB 003).

Immunohistochemical studies
Antibody
- Supplier
- Catalog #
- Validation
- RRID
- Reference

CP13
- Peter Davies gift
- n/a
- Validation not found in original reference
- AB_2314223

- Jicha et al., J Neurosci, 1999
Herkovits et al., Neurobiol Dis, 2006
https://www.alzforum.org/antibodies/tau-phos-ser202-cp13

Ab39
- Shu-Hui Yen gift
- n/a
- They are detected in tissues that have been in formalin for as long as 17 years. None of our anti-ANT antibodies, except Ab 322, reacted with HeLa cells (Figure 7) or cultured rat cerebellar cells, which contain astrocytes and fibroblasts. Incubation of monoclonal supernatant for 1 hour at room temperature with 1 mg of microtubule preparation or brain filament proteins does not remove the anti-tangle activity, whereas incubation with 10 jig of SDAT brain homogenate with the antibody removes partially the anti-tangle activity. Complete absorption could be achieved with 100 jig of SDAT brain homogenate (Figure 9). Recognizes only SDS-insoluble proteins from SDAT excluded at the top of the gel.
- n/a
- Yen et al., Am J Pathol, 1984

33.1.1
- Pritam Das gift
- n/a
- Validation not found in original reference
- n/a
- Levites et al., J Clin Invest, 2006
Kim et al., J Neurosci, 2008

GFAP
- Biogenex
- MU020-UC
- BioGenex has conducted studies to evaluate the performance of the antibody with BioGenex detection systems and accessories. The antibodies have been found to be sensitive and show specific binding to the antigen of interest with minimal to no binding to non-specific tissues or cells.
- AB_2847821
- https://store.biogenex.com/us/anti-gfap-clone-ga-38.html
CD34
- Abcam
- Ab81289
- Tested applications Suitable for: WB, IHC-P, ICC/IF, IP, IHC-Fr, Flow Cyt Species reactivity Reacts with: Mouse, Rat, Human Predicted to work with: Sheep, Dog, Pig, African bush elephant
- AB_1640331
- https://www.abcam.com/cd34-antibody-ep373y-ab81289.html

CD68
- Dako
- M0814
- The antibody was clustered as anti-CD68 at the Fourth International Workshop and Conference on Human Leucocyte Differentiation Antigens held in Vienna in 1989 (5). SDS-PAGE analysis of immunoprecipitates formed between the antibody and 125I-labeled lysates from human spleen with B-cell lymphoma rich in macrophages shows reaction with a 110 kDa polypeptide, corresponding to CD68 (4). In Western blotting of extracts of lung, spleen and U937 cells, diffuse 110, 70 and 40 kDa bands were detected when using reducing conditions. Under non-reducing conditions the spleen extract showed an additional 220 kDa band (4). See package insert for reference(s).
- AB_2314148
- https://www.agilent.com/en/product/immunohistochemistry/antibodies-controls/primary-antibodies/cd68-(concentrate)-76535
Immunofluorescence studies
Primary Antibody
- Supplier
- Validation
- RRID
- Reference

GFAP
- ABCAM ab33922
- Tested applications Suitable for: ICC/IF, WB, IHC-P Unsuitable for: Flow Cyt or IP Species reactivity Reacts with: Rat, Human
- AB_732571
- https://www.abcam.com/gfap-antibody-ep672y-ab33922.html

MAP2
- ABCAM ab32454

- Tested applications Suitable for: IHC-P, ICC, WB Species reactivity Reacts with: Mouse, Rat, Human Predicted to work with: Goat, Cat, Lizard
- AB_776174
- https://www.abcam.com/map2-antibody-neuronal-marker-ab32454.html
IBA1
- ABCAM ab178847
- Tested applications Suitable for: IHC (PFA fixed), IHC-P, WB, IP, ICC/IF Species reactivity Reacts with: Mouse, Rat, Human
- AB_2832244
- https://www.abcam.com/iba1-antibody-epr16589-ab178847.html

CD68
- Cell Signaling 76437
-Tested applications Suitable for: F, IF-IC, IHC-P Species reactivity Reacts with: Human
- AB_2799882
- https://www.cellsignal.com/products/primary-antibodies/cd68-d4b9c-xp-rabbit-mab/76437

CD34
- ABCAM ab81289
- Tested applications Suitable for: WB, IHC-P, ICC/IF, IP, IHC-Fr, Flow Cyt Species reactivity Reacts with: Mouse, Rat, Human Predicted to work with: Sheep, Dog, Pig, African bush elephant
- AB_1640331
- https://www.abcam.com/cd34-antibody-ep373y-ab81289.html
OLIG2
- ABCAM ab109186
- Tested applications Suitable for: ICC, WB, IHC-P Species reactivity Reacts with: Mouse, Rat, Human
- AB_10861310
- https://www.abcam.com/olig2-antibody-epr2673-ab109186.html

Tau E1
- Leonard Petrucelli gift
- In heat-stable preparations from human brains, antibody E-1 bound to multiple bands (Figure 1 A). The antibody E-1-positive proteins migrated in a region corresponding to microtubule-associated protein tau. The staining pattern was comparable to that displayed with Tau-1 (Figure 1A) and other anti-tau antibodies. Preimmune serum did not react with brain proteins. The antibody E-1-reactive proteins from animal brains were not found in the perchloric acid-soluble supernatant. Instead they were detected in the acid precipitates. Absorption of Ab E-1 antiserum with E-1 peptide removed the antibodies responsible for the staining of 60- to 68-kd proteins on immunoblots
- AB_2819185
- Crowe, Am J Pathol, 1991
Tau pS396
- Abcam 109390
- Tested applications Suitable for: Dot blot, IHC-Fr, WB, IP Unsuitable for: ICC/IF Species reactivity Reacts with: Mouse, Rat, Human
- AB_10860822
- https://www.abcam.com/tau-phospho-s396-antibody-epr2731-ab109390.html

Tau E178
- Abcam 32057
- Tested applications Suitable for: IHC-P, IHC-Fr, WB, IP Unsuitable for: Flow Cyt Species reactivity Reacts with: Mouse, Rat Predicted to work with: Cow, Human
- AB_778254
- https://www.abcam.com/tau-antibody-e178-ab32057.html

IgG2a
- R&D MAB003
- Isotype control
- AB_357345
- https://www.rndsystems.com/products/mouse-igg-2a-isotype-control_mab003

Human research participants

Policy information about studies involving human research participants

Population characteristics	Postmortem brain tissue from neuropathologically diagnosed Alzheimer's disease and nondemented controls were obtained from the Mayo Clinic brain bank. The metric used to investigate RNA quality, DV300, ranged from 74 to 96. This assesses the proportion of intact RNA fragments with greater than 300 nucleotides. After tissue was harvested at autopsy, samples were stored at -80ºC. Alzheimer's disease cases and controls were both males and females, ranging in age at death from 55 to 103 years.
Recruitment	Brains were derived from the Mayo Clinic brain bank, which receives brains from all over the United States.
Ethics oversight	Brains were donated with the consent of the individual and/or their next-of-kin. The research performed on postmortem samples was approved by the Mayo Clinic Research Executive Committee (IRB17-007585/ Bio00015595).

Note that full information on the approval of the study protocol must also be provided in the manuscript.
